# Chemical adherence testing in the clinical management of hypertension: a scoping review

**DOI:** 10.3389/fphar.2024.1452464

**Published:** 2024-11-06

**Authors:** Louise Rabbitt, James Curneen, Michael Conall Dennedy, Gerard J. Molloy

**Affiliations:** ^1^ Department of Pharmacology, School of Medicine, University of Galway, Galway, Ireland; ^2^ Galway University Hospital, Saolta Healthcare Group, Galway, Ireland; ^3^ School of Psychology, University of Galway, Galway, Ireland

**Keywords:** hypertension, adherence-compliance-persistance, chemical adherence testing, mass spectrometry, blood pressure, antihypertensive

## Abstract

**Background:**

Despite growing use, questions remain surrounding the utility, acceptability and feasibility of chemical adherence testing (CAT) as part of hypertension management in clinical practice.

**Objectives:**

This scoping review aimed to (i) identify and summarise studies using CAT in hypertension management, and (ii) describe and critically evaluate how CAT is currently being used in the clinical management of hypertension.

**Eligibility criteria:**

Peer-reviewed and published studies in English, reporting original research in any setting, with any study design, were included. Search concepts included hypertension, medication adherence, CAT, and their synonyms.

**Sources of evidence:**

Searches were carried out using Ovid Medline, EMBASE, and PsycInfo (EBSCO), alongside manual searching of reference lists. Using Covidence software, we screened titles and abstracts, followed by full-text articles. Data from the included articles were tabulated and summarised.

**Results:**

Of the 618 studies identified, 48 were included. The studies cover diverse clinical settings, and were mostly observational in design. 7 studies reporting adherence analyses within clinical trials for hypertension therapies. The use of theoretical frameworks to guide reporting was rare, and there was considerable variation in key terminology and definitions, most notably in the definition of adherence.

**Conclusion:**

The current body of evidence demonstrates considerable variability in the approach to implementing CAT for hypertension management in clinical practice, and a paucity of randomised controlled trials to evaluate its impact. Future research could (i) adopt a cohesive theoretical framework including clear operational definitions to standardise the approach to this important topic; (ii) further explore the impact of CAT on clinical outcomes using RCTs.

## Introduction

Using medicines as prescribed can be a particular challenge in those common chronic conditions that are asymptomatic (Burnier and Egan, 2019). The pain-relief provided by long-term analgesia use, e.g., paracetamol, or the reduction in respiratory symptoms provided by some anti-inflammatory agents, e.g., corticosteroids, can provide a potent means of supporting patient initiation and persistence with long-term therapies (Rottman et al., 2017). In these instances, patients directly experience the benefits of using medicines and the aversive consequences of prematurely terminating medicine use. However, the most frequently used medicines, particularly in older adulthood, are those used for diseases where there is no discernible experience of an illness, such as hypertension (Choudhry et al., 2022).

Hypertension represents the greatest burden of non-communicable disease associated morbidity and mortality globally with a worldwide adult prevalence of disease estimated at 31% and affecting 1.39 billion individuals (Forouzanfar et al., 2015; Mills et al., 2016). Internationally, blood pressure remains above target in 63% of all diagnosed hypertensive patients in high-income western countries (Zhou et al., 2019). Several factors contribute to poor blood pressure control including undiagnosed or unrecognised secondary hypertension, so-called treatment resistant hypertension, physician inertia, and non-adherence to anti-hypertensives (Bunker et al., 2011; Durand et al., 2017; Hayes et al., 2019; Kjeldsen et al., 2015).

Adherence to antihypertensive drug (AHD) therapy is central to sustained control of blood pressure, reducing clinic visits and reducing complications of undertreated hypertension (Berra et al., 2016; Hill et al., 2011; Mazzaglia et al., 2009). Moreover, identifying non-adherence in patients who are not meeting BP targets could help providers avoid over-prescription and unnecessary investigation, and to prioritise patients who require more detailed investigation for secondary causes of hypertension, thereby having substantial clinical and economic impact (Schoonhoven et al., 2018).

Hypertension care providers report having little time and few tools to support detecting and improving adherence in their patients (Burnier et al., 2021). Objective assessment of adherence using chemical adherence testing, where available, is recommended by the 2023 European Society of Hypertension (ESH) guidelines for the management of arterial hypertension and the 2024 European Society of Cardiology (ESC) Guidelines for the management of elevated blood pressure and hypertension, and has been described as one of the most reliable methods for assessing adherence (Hayes et al., 2019; Tomaszewski et al., 2014; Curneen et al., 2022; Mancia et al., 2023; McEvoy et al., 2024; Wunder et al., 2019).

High performance liquid chromatography tandem mass spectrometry (LC-MS/MS), can measure anti-hypertensives and their metabolites within patient urine or blood samples, providing point-in-time estimation of anti-hypertensive adherence. LC-MS/MS of urine is usually employed as a qualitative method, describing presence or absence of drugs only, and results are influenced by inter-drug and inter-individual differences in pharmacokinetics (Berra et al., 2016; Wunder et al., 2019). Urine LC-MS/MS analysis can also detect drug metabolites which may be detectable for longer periods of time than the parent drug itself. In this way, urine analysis tends to refer to a longer period of time than serum analysis. LC-MS/MS analysis of serum may provide a more accurate point-in-time estimation of adherence as it allows for quantitative assessment to determine the drug level, which can be used to optimise drug dosage or estimate the time since last intake (Ritscher et al., 2020). Analysis of oral fluids and hair have also been suggested though neither is currently commonly used (Sharma et al., 2023; Lauder et al., 2020).

LC-MS/MS, has several advantages over other methods of adherence assessment. Self-report has been shown to correlate poorly with direct or objective methods of adherence measurement (Osterberg and Blaschke, 2005). Pharmacy dispensing records may not adequately reflect adherence if prescription data are not captured from all potential sources or patients do not take the dispensed medications (Ruzicka et al., 2019). Electronic pill boxes may not always be available and are less acceptable and feasible for those on multiple medicines, such as people with resistant hypertension (RH) (El Alili et al., 2016; Van Onzenoort et al., 2012). Directly Observed Therapy (DOT) combined with ambulatory BP monitoring (ABPM) has also been successfully employed (Hjørnholm et al., 2019). It may present feasibility challenges as it requires resources for monitoring, given the potential to cause symptomatic hypotension (Ruzicka et al., 2019).

However, despite growing consensus that chemical adherence testing (CAT) represents a potentially valuable tool in hypertension management (Mancia et al., 2023; Wunder et al., 2019), particularly in hypertension which has proven difficult to treat, the optimum manner of its use remains unclear. A disparate literature on CAT use in hypertension is developing where agreement on key terminology, definitions and methods is only beginning to emerge over the last 5 years (Wunder et al., 2019). There is a pressing need, therefore, to carry out evidence syntheses, as relevant studies have straddled multiple basic science and clinical literatures.

As distinct from systematic reviews, scoping reviews allow for a broader focus and present results in descriptive formats that highlight what kinds of evidence exist, where there are evidence gaps, and the quality of the existing evidence (Nyanchoka et al., 2019; Arksey and O’Malley, 2005). Scoping reviews are also recommended when there is a need to clarify the key constructs and operational definitions employed in an area of research, to examine the ways in which research in an emerging area is being conducted and to identify the factors associated with a specific concept (Munn et al., 2018; Noone et al., 2021).

For these reasons, we elected to conduct a scoping review to assess the characteristics of research in which chemical adherence testing is implemented in the clinical management of hypertension. The aims of this review were to (i) identify and summarise studies using CAT in hypertension management, and (ii) describe and critically evaluate how CAT is currently being used in the clinical management of hypertension. We report here our findings with reference to the PRISMA-ScR (Preferred Reporting Items for Systematic Reviews and Meta-Analyses extension for Scoping Reviews) checklist (Tricco et al., 2018).

## Methods

### Protocol and registration

The protocol for this scoping review was registered prior to data extraction on Open Science Framework Registries (Rabbitt et al., 2024).

### Research question

To address our aims, we formulated the following research questions:

1. What are the characteristics of research methods on the implementation of CAT for anti-hypertensive pharmacotherapy in clinical practice?
2. What characteristics of CAT implementation can be discerned (e.g., clinical setting, what type of CAT, where in the patient journey)?

### Information sources and search strategy

The search was conducted with the assistance of a health sciences librarian. Synonyms for three core concepts were iteratively tested: *medication adherence, chemical adherence testing,* and *hypertension.* Three electronic databases were searched from inception to April 2024: MEDLINE (Ovid); EMBASE; and PsycINFO (EBSCO). These databases were chosen given their relevance to the core concepts. In addition, we manually screened the reference lists of review articles identified during screening for relevant references. We used standardised medical subject headings and subject headings provided by the chosen databases. Synonyms were joined by the Boolean operator *OR*; thereafter, the search strings for each concept were combined with the Boolean operator *AND*.

#### Search concepts

1. Medication adherence
2. Chemical adherence testing
3. Hypertension

#### Search terms (examples–for full search strategy see Supplemental Data Sheet 1)

1. Treatment adherence and compliance; patient compliance; medication adherence
2. Chemical adherence testing; drug monitoring; therapeutic drug monitoring; mass spectrometry
3. Hypertension; blood pressure; antihypertensive drugs

### Eligibility criteria

The inclusion and exclusion criteria are shown in Table 1.

**TABLE 1:** Eligibility criteria.

Inclusion criteria	Exclusion criteria
⁃Prospective studies reporting original research published in biomedical journals ⁃Systematic reviews, meta-analyses ⁃Letters to the editor, guidelines, policy documents ⁃Any study design ⁃English language	⁃Non-peer-reviewed data ⁃Review articles, opinion articles ⁃Studies demonstrating the technical procedure of CAT without use in a clinical population

### Data sought

The types of data collected included clinical data on people with a diagnosis of hypertension, taking antihypertensive medication(s), in any healthcare setting. Methods and outcomes of interest were CAT, with or without comparisons with other methods of measuring medication adherence.

### Study selection and synthesis

All identified records were imported into Covidence, a web-based collaboration software platform that streamlines the production of systematic and other literature reviews (Veritas Health Innovation Melbourne Australia, 2024). Duplicates were removed and the titles and abstracts of the remaining records were screened for eligibility by at least one of the authors. Uncertainty or conflict was resolved by discussion until consensus was reached. Full-text articles were then screened independently by two of the authors. Again, conflict or uncertainty were resolved through discussion until consensus was reached. The Covidence data extraction and critical appraisal templates were adapted to address the aims of this review.

The following data were extracted and tabulated:

1. General information: Authors, publication year, country of origin, clinical setting, study aim and study design
2. Participant information: Diagnoses, basic demographic details, number of participants enrolled,
3. CAT details: substrate and method for CAT, whether participants were informed in advance of CAT, whether CAT results were fed back to participants, definition of adherence, phase of adherence targeted, CAT carried out once or on multiple occasions.
4. Results: Key findings with respect to adherence, key findings with respect to blood pressure control or other pertinent clinical outcomes.

### Critical appraisal

Depending on the study design, the following quality appraisal tools were applied to the included studies: the Joanna Briggs Institute Critical Appraisal Checklist for analytical cross-sectional research (Moola et al., 2020), and the Cochrane Risk of Bias Tool (Sterne et al., 2019) for Randomised Controlled Trials. Quality assessment was carried out by one reviewer and checked by another. The major confounders considered included the potential for white-coat adherence if participants were informed in advance of the intention to carry out CAT. In addition, we assessed whether studies published in 2018 or later included the four minimum reporting criteria set out by the European Society for Patient Adherence (ESPACOMP) in the ESPACOMP Medication Adherence Reporting Guideline (EMERGE) (De Geest et al., 2018). These guidelines represent an attempt to improve the reporting in adherence research by providing a theoretical framework.

## Results

**FIGURE 1:**
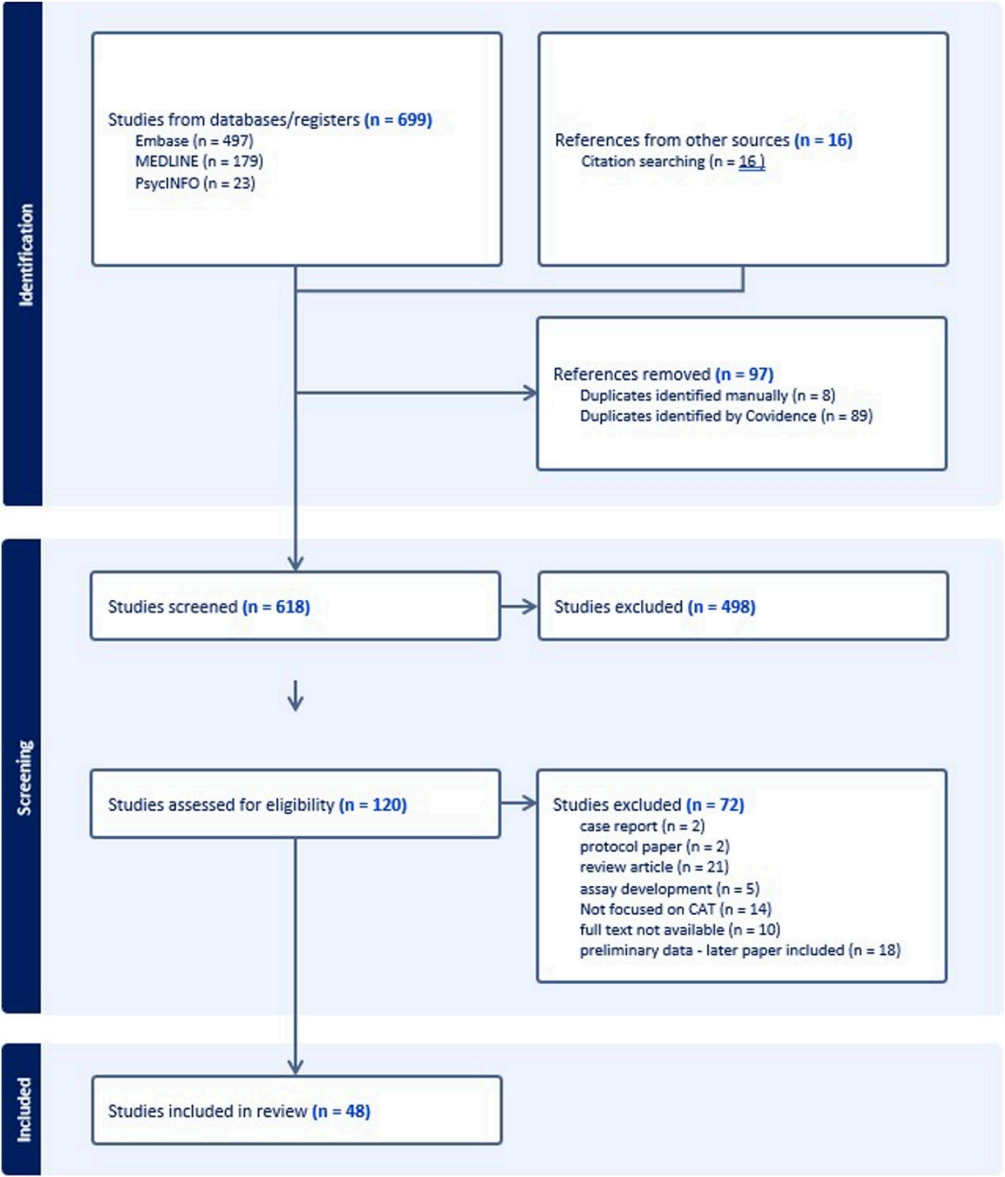
PRISMA flow diagram.

We identified 699 records, of which 683 (97.7%) were identified through database searches, and 16 (2.3%) through manual searches of reference lists in the review articles. After removal of duplicates, we screened titles and abstracts of 618, and the remaining 120 were assessed for eligibility through full-text review. Of these 120, 72 were excluded for the reasons shown in Figure 1, and 48 were included in the scoping review.

### Research question 1: characteristics of sources of evidence

**FIGURE 2:**
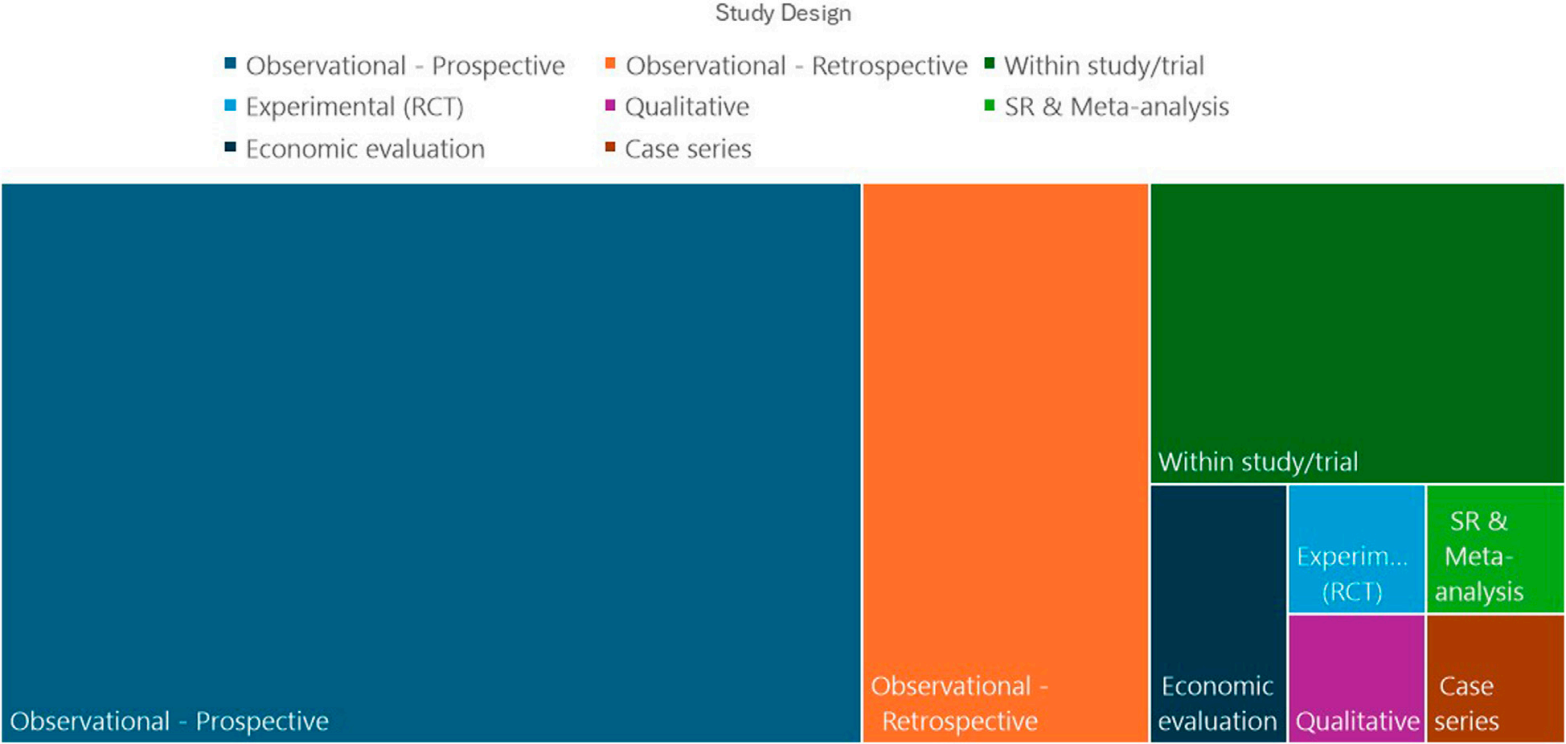
Designs of included studies.

Table 2 shows the characteristics of the included studies. Most of the reviewed studies (44/48) were published within the past 10 years. Most (46/48) of the studies originated from North America and Europe. Figure 2 shows the distribution of study designs. The majority of included studies were observational, and despite several authors pointing out the need for randomised controlled trials (RCT) to delineate the contribution of CAT to optimising hypertension management (Osula et al., 2022), only one RCT was identified which directly examined the effect of CAT (Valgimigli et al., 2019).

**TABLE 2:** Characteristics of included studies.

Author, year	Country	Setting	Primary aim of study	Study design	Total number of participants
Peeters 2024	Netherlands	Vascular, cardiology and nephrology hospital departments	To determine whether a CAT intervention combined with feedback using a communication tool leads to a decrease in resistant hypertension	RCT	100
Kario 2023	Japan	Clinical trial	Post-hoc analysis of stored urine samples in order to evaluate medication adherence	Post-hoc analysis within RCT	58
Kustovs 2023	Latvia	University hospital	To establish a target population of patients with possible changes in drug compliance despite the wide range of fixed-dose combinations and in whom it would be useful to determine the concentration of amlodipine in the blood	Prospective cross sectional study	81
Seleznev 2023	Russia	Regional Clinical Cardiological Dispensary	To test the concentration of antihypertensive drugs in patients with uncontrolled and controlled arterial hypertension	Cohort study	46
Curneen 2023	Ireland	Specialist hypertension clinic	To compare patient reported antihypertensive adherence with objective evidence using mass spectrometry spot urinalysis	Prospective cohort study	73
Peeters 2023	Netherlands	Hospital nephrology and vascular clinics	To determine the adherence to antihypertensive drugs in patients visiting the nephrology and vascularoutpatient clinics using CAT	Prospective cross sectional study	142
Osman 2023	United Kingdom	University hospital renal clinic	To demonstrate and highlight the usefulness of CAT to determine the prevalence of nonadherence to cardio-metabolic medications in patients attending routine renal clinics	Prospective cross sectional study	106
Bourque 2023	Canada	Multiple	To report on the overall prevalence of nonadherence in the apparent treatment resistant hypertension population and the quantitative contributions to nonadherence based on different methods of assessment, with an emphasis on attempting to explain the heterogeneity of the data	Systematic review and meta-analysis	71,353
Georges 2022	Belgium; Italy	Cardiology Dept; Hypertension Expert Centre	To document associations between psychological profile, drug adherence, and severity of hypertension in a representative sample of patients with apparent treatment resistant hypertension, using controlled hypertensive patients as the comparator	Prospective cross sectional Study	144
Sheppard 2022	United Kingdom	Primary care	To investigate whether it is feasible to collect urine samples in a primary care setting and analyse them using the LC-MS/MS method to measure adherence to antihypertensive medications	Prospective cohort study	191
Groenland 2022	Netherlands; United Kingdom	Hospital outpatient clinics	To develop and externally validate a screening tool, based on easy to collect clinical variables, to estimate the probability of non-adherence in patients with uncontrolled hypertension	Cross sectional study	735
Peeters 2022	Netherlands	Clinical trial	To illustrate the importance and difficulties that can arise using a three-step approach to medication adherence	Case series within RCT	3
Osula 2022	United States	Internal Medicine and Cardiology Clinics in a large urban safety net health system	To compare the sensitivity, specificity, and predictive values of pharmacy fill data measures of adherence obtained from a nationwide prescribing database against CAT in detecting nonadherence with cardiovascular medications in patients with uncontrolled hypertension in the safety net health system	Prospective cross sectional study	77
Wang 2021	China	Hospital	To ensure drug compliance during a catheter-based therapy for treatment of hypertension	Cross sectional study	92
Buffolo 2021	Italy	Hypertension unit of university hospital	To evaluate the aldosterone:renin ratio changes, before and after ARB/ACEi initiation, as a means to assess adherence to ARB/ACEi prescription	Prospective cohort study	40
Beernink 2021	Netherlands	Hospital/Trial	To assess the prevalence of nonadherence to oral antidiabetics, antihypertensives, and statins within a cohort study of type 2 diabetes patients managed in a specialist setting using CAT	Prospective cohort study	457
Schäfer 2021	Germany	Hypertension clinic in university medical centre	To analyse patients’ suitability for baroreceptor activation therapy and reasons for non-eligibility in patients with apparently resistant hypertension	Retrospective cross sectional study	75
Lauder 2021	Germany	Emergency Department of University Medical Centre	To identify treatment-related and psychosocial characteristics, including anxiety, depression, and health literacy, associated with nonadherence to BP-lowering medication among patients with previously diagnosed hypertension presenting with hypertensive urgencies at an emergency department	Prospective cross sectional study	104
Schesing 2020	United States	Outpatient clinics in an integrated health system which provides care for a low- income, uninsured population	To explore patients’ and providers’ knowledge, attitudes, beliefs and concerns about using a blood test to monitor medication adherence and how best to introduce and use CAT in a respectful, patient-centred way	Qualitative study	21
Wunder 2019	Belgium, Netherlands	Clinical trial	To give an impression on the reliability of adherence assessment during a trial	Analysis within randomised parallel group trial	18
Pelouch 2019	Czechia	Hospital clinic	To assess the drug non-adherence in stable CHF patients using serum drug levels monitoring	Prospective cross sectional study	81
Hayes 2019	Ireland	Primary care	To examine the feasibility of establishing non-adherence to medication using mass spectrometry urine analysis in primary care	Prospective cross sectional study	235
deJager 2018	Netherlands	Clinical trial	Post-hoc analysis to explore possible determinants of nonadherence in treatment resistant hypertension, within a trial to assess the effect of renal denervation on BP 6 months after treatment compared to usual care in patients with resistant hypertension	Substudy of open label RCT	98
vanSchoonhoven 2018	Netherlands, United Kingdom	N/A	To model the cost-effectiveness of performing LC-MS/MS-based analyses in improving adherence in patients with hypertension	Economic Evaluation	N/A
Sandbaumhüter 2018	Switzerland	Hypertension clinic	To use CAT to verify drug adherence during routine laboratory screening for PA and check for potential drug bias of the results	Prospective cohort study	24
Sutherland 2018	United States	Emergency Department	To validate a serum-based LC-MS/MS assay to simultaneously quantify 263 medications used for acute and chronic conditions	Prospective cross sectional study	
Avataneo 2018	Italy	Hypertension Unit	(i) To describe the prevalence of nonadherence in a representative sample of Italian patients with resistant hypertension using therapeutic drug monitoring on plasma samples. (ii) To determine clinical and/or demographic parameters associated with poor therapeutic adherence	Prospective cross-sectional	50
Petit 2018	Belgium	Cardiology department in an academic hospital	(i) To document the level of adherence to drug treatment in a sample of patients with aTRH using a direct evaluation method (ii) to explore the relations between psychological profile assessed by a broad array of validated questionnaires, adherence to antihypertensive medications as measured by LC-MS/MS, and degree of drug treatment-resistance evaluated by on-treatment 24-h ambulatory BP measurement	Prospective cross sectional study	35
Gupta 2017 (Burnier and Egan, 2019)	United Kingdom and Czech Rep	UK: samples processed by University Hospital of Leicester, from 15 UK sites. Czech Rep: Hypertension Unit of University Hospital	To detect nonadherence and explore its association with the main demographic and therapy related factors in patients with hypertension	Retrospective cross sectional study	1,348
Jones 2017	South Africa	Referral hypertension clinic	To determine whether monitoring plasma amlodipine concentrations and inhibition of angiotensin-converting enzyme (ACE) can be adjunct adherence tools	Prospective cross sectional study	100
Hamdidouche 2017	France	Academic medical center specialty hypertension clinic	To assess the prevalence of drug nonadherence under routine clinical conditions, the factors associated with nonadherence, and the impact of directly measured nonadherence on BP control	Prospective cohort study	174
Gupta 2017	United Kingdom and Czech Rep	Hospital blood pressure clinic	To examine the potential therapeutic applications of biochemical screening for the presence of antihypertensive medications in bodily fluids	Retrospective cohort study	331
Kocianova 2017	Czechia	Outpatient hypertension unit in university hospital	To evaluate the ratio of the non-adherent patients according to plasma levels of beta blockers and to study the relation of the plasma levels to patients’ office heart rate	Retrospective cross sectional study	106
McNaughton 2017	United States	Emergency department at an academic hospital	To test the hypothesis that higher antihypertensive medication adherence, biochemically assessed by a LC-MS/MS blood assay, would be associated with lower BP in the ED setting after adjusting for multiple patient demographic and clinical factors	Prospective cross-sectional study	261
Bohlender 2017	Switzerland	Hospital hypertension clinic	(i) To verify drug adherence during routine laboratory screening for PA and (ii) check for potential drug bias of the results	Prospective observational pilot study	24
Schmieder 2016	Germany	Clinical research center, dept of nephrology and hypertension	To report adherence rates at baseline and at 6 months after renal denervation and the relationship between adherence and BP measurements in patients with resistant hypertension	Analysis within prospective clinical trial	79
Patel 2016	United Kingdom	Specialist hypertension centre	To examine the extent to which integration of CAT into the diagnostic pathway may affect the ultimate eligibility rates for renal denervation	Retrospective analysis	34
Beaussier 2015	France	Clinical trial	To assess the influence of medication adherence on BP control and target organ damage in a pre-specified analysis of a published trial comparing sequential nephron blockade or sequential renin-angiotensin system blockade in patients with resistant hypertension	Randomised controlled trial	164
Ewen 2015	Germany	Clinical research	To determine the individual intake of antihypertensive drugs in patients with resistant hypertension undergoing renal denervation	Prospective cohort study	100
Florczak 2015	Poland	Clinical research	To evaluate adherence to therapy in patients with resistant hypertension by determining serum antihypertensive drug levels with the use of LC-MS/MS	Cross sectional study	36
Velasco 2015	United States	Specialist hypertension referral clinic	(i) To determine the relationship between primary aldosteronism (PA) prevalence and medication adherence. (ii) To build a decision analysis model to test the cost effectiveness of a CAT-guided approach for PA screening in patient swith apparent TRH, compared with a nonselective approach	Cross sectional study; Economic Evaluation	78
Tomaszewski 2014	United Kingdom	Specialist clinical hypertension centre	To report HPLC-MS/MS analysis of spot urine samples in hypertensive patients attending a specialist clinical hypertension centre	Retrospective cross sectional study	208
Rosa 2014	Czechia	Hypertension centre	To assess the proportion of patients eligible for renal denervation	Cohort study	205
Brinker 2014	United States	Hospital hypertension clinic	(i) To assess the impact of CAT in optimising BP control in patients with resistant hypertension. (ii) To establish cost-effectiveness of CAT	Retrospective study	56
Jung 2013	Germany	Nephrology outpatient department	To use CAT to determine the impact of adherence in patients with apparent resistant hypertension and to assess possible factors related to drug therapy adherence	Retrospective chart review	76
Strauch 2013	Czechia	Hypertension unit within university hospital	To assess the prevalence of pseudo-resistance caused by noncompliance with treatment among patients with severe resistant hypertension and to analyze the contributing factors	Cohort study	339
Ceral 2011	Czechia	Hypertension clinic	To evaluate serum levels of prescribed antihypertensive drugs in individuals with difficult-to-control arterial hypertension	Retrospective cross sectional study	84
Azizi 2006	Multicentre: 16 countries in Europe ad North Africa	General Practice	To assess patients compliance with ACE inhibitor treatment in the DIABHYCAR study	Analysis within randomized, double blind, parallel-group trial	1,871

Seven of the included studies reported adherence analyses carried out within clinical trials (Azizi et al., 2006; Ewen et al., 2015; Beaussier et al., 2015; Kario et al., 2023; de Jager et al., 2018; Ceral et al., 2011), of which five were clinical trials of renal denervation (RDN). Of these studies relating to RDN, three carried out CAT as pre-specified analyses in the trial design (Azizi et al., 2006; Ewen et al., 2015; Johnson and Hennessy, 2019), and 2 as *post hoc* analyses (Kario et al., 2023; de Jager et al., 2018). In addition, three observational studies reported adherence rates in patients undergoing RDN, or screening for RDN (de Jager et al., 2018; Patel et al., 2016; Ceral et al., 2011). One systematic review and meta-analysis is included (Bourque et al., 2023). This aimed to establish the overall prevalence of nonadherence in resistant hypertension and compare direct (such as CAT) and indirect (such as pill counting) methods of adherence assessment. The authors found that in 42 studies including 71,353 patients, indirect methods reported less than half the rates of non-adherence compared to direct methods. One qualitative study used interviews with patients and providers and discussion with a community advisory panel to explore attitudes towards using CAT in the clinical management of hypertension (Schesing et al., 2020).

The economic impact of CAT is a growing concern in the literature and will be of interest to those managing and designing clinical services for hypertension. Two studies explored the cost-effectiveness of CAT, in view of the potential for CAT to (i) rationalise diagnostic decision-making and investigations, and (ii) improve BP control and thereby clinical outcomes for patients (Schoonhoven et al., 2018; Velasco et al., 2015).

#### Included populations

The majority of studies took place in hospital-based secondary or tertiary care settings, with just 3 reported from primary care. Some studies deployed CAT in a targeted way, according to specified clinical criteria such as aTRH, or at the discretion of the treating physician (Florczak et al., 2015; Groenland et al., 2022; Gupta et al., 2017a; Schäfer et al., 2021). Others applied CAT in a non-discriminatory manner, to all patients attending a given service. Ten studies explicitly stated that patients with secondary hypertension were excluded but the manner of screening for secondary hypertension was not always detailed. Eight studies only included participants who reported having taken their medicines as prescribed and excluded those who reported non-adherence (Osula et al., 2022; Ewen et al., 2015; Velasco et al., 2015; Avataneo et al., 2018; Strauch et al., 2013; Jung et al., 2013; Kocianova et al., 2017; Gupta et al., 2017b); this has important implications when considering the rates of false positive CAT results. Supplemental Table 2 shows the characteristics of participants in the included studies.

### Research question 2: characteristics of CAT implementation

#### Methods of CAT

The characteristics of CAT used in the studies is summarised in Supplemental Table 1. Of the 45 included primary quantitative studies, 44 studies used mass spectrometry of either urine (22 studies), serum, dried blood spot, or a combination of samples, to directly detect AHDs or their metabolites. LC-MS/MS was most commonly used but gas chromatography-mass spectrometry and spectrofluorometry were also used (Schäfer et al., 2021; Brinker et al., 2014). Five studies used alternative methods, either alone or in conjunction with LC-MS/MS. These were chiefly assays of the renin-angiotensin-aldosterone axis, such as serial aldosterone to renin ratio measurement (Buffolo et al., 2021), serum Z-FHL/HHL (z-phenylalanine-histidine-leucine/hippuryl-histidine-leucine) ratio (Jones et al., 2017), or urine AcSDKP/creatinine ratio (Beaussier et al., 2015; Hamdidouche et al., 2017). These alternative methods may be useful to providers in situations where LC-MS/MS laboratory analysis is not available. In 27% (12/45) of studies, CAT was performed on more than one occasion, while for the remainder it was performed only once.

#### Interpretation and application of CAT results

There was considerable variation in the definition of adherence. Adherence was variously considered a dichotomous, categorical or continuous variable. Of the studies using LC-MS/MS, three studies considered a participant “fully” adherent if at least 80% of their prescribed AHDs were found to be present (de Jager et al., 2018; Lauder et al., 2021; Schmieder et al., 2016), while the others required 100% concordance to consider someone adherent. Similarly, while most studies differentiated between “partial” and “complete” non-adherence, ten studies considered a participant non-adherent if there was any discrepancy between their prescribed AHDs and the CAT results (Osula et al., 2022; Ewen et al., 2015; Florczak et al., 2015; Brinker et al., 2014; Ceral et al., 2011; Gupta et al., 2017b; Pelouch et al., 2019; Sheppard et al., 2022; Osman et al., 2023; Beernink et al., 2021). Some studies attempted to address the limitations of CAT by combining it with other methods of adherence testing, for example, Beaussier (2015) uses an adherence scoring system which combines two CAT modalities with self-report and pill counting (Curneen et al., 2022; Beaussier et al., 2015). Six (13%) studies described reporting back the results of CAT to patients, while the remainder either didn’t provide participants with their results, or did not state whether participants received the results of the CAT. The majority of studies were descriptive cross-sectional studies which did not measure longer-term outcomes for patients. Just 4 studies (9%) reported on the impact that CAT had on clinical outcomes for patients (Velasco et al., 2015; Brinker et al., 2014; Gupta et al., 2017b; Valgimigli et al., 2019).

### Critical appraisal results

We applied the 4 minimum reporting criteria from the EMERGE guidelines to the included studies published after the guidelines’ publication in 2018 (2019 or later; 22 studies). Of the 22 studies, just 2 (9%) of them included the four minimum reporting criteria set out by the EMERGE guidelines (Groenland et al., 2022; Buffolo et al., 2021). One further study met three of the four criteria (Curneen et al., 2022), while the remaining 19 (86.4%) did not include any of the minimum reporting criteria. It should be noted that most papers did detail the performance of the CAT measure with regard to its validity and reliability but did not consider these factors in reference to the phase(s) of adherence studied. The judgements for each study are included in Supplemental Table 3.

The JBI tool for assessing the quality of cross-sectional studies was applied to 40 studies. For 32 (80%) of these studies, the inclusion criteria were clearly defined, and in 31 (77.5%) the subjects and setting were described in detail. 35 (87.5%) studies used objective, standard criteria when measuring the condition (BP in this case). Confounding variables were identified in 30 (75%) studies, and of these, 13 (43.3%) described a strategy for dealing with these confounding factors. The statistical analysis was considered to be appropriate for 33 (82.5%) of studies. The judgements for each study are presented in Supplemental Table 4.

For the only included RCT which directly assessed the effect of CAT, the Cochrane risk of bias (RoB) tool revealed some concerns, primarily around the unblinded intervention, and the fact that some patients developed an aversion to ambulatory blood pressure monitoring, necessitating the use of alternative BP measures. Moreover, this RCT encountered some difficulties in recruitment and study visits due to the COVID-19 pandemic (Valgimigli et al., 2019).

## Discussion

The aim of this review was to identify studies using CAT in hypertension and to describe and critically evaluate how CAT is currently being used in the clinical management of hypertension. We found that the use of CAT in hypertension is gaining significant research interest. We found that research on CAT in hypertension is mostly published in high-income countries, focussed on treatment-resistant hypertension in secondary or specialist healthcare settings, and usually observational in design. Few studies measured the impact that performing CAT has on clinical outcomes for patients, such as BP control. This means that increasing calls for CAT to form part of routine clinical care in hypertension are underpinned by largely observational data. There are relatively few randomised trials to inform CAT use. One recent RCT, published outside the time limit for this review, found no effect of CAT on BP control or adherence, though it was underpowered (Peeters et al., 2024). A number of challenges have been demonstrated with conducting RCTs in the area of adherence (Muntner and Tanner, 2024). The variability in BP control and adherence over time impedes the identification of patients suitable for recruitment. Patients most challenged by adherence may be less likely to be included in trials because of non-attendance, low literacy, low motivation, language barriers, or other psychosocial challenges. Hawthorne effects may influence medication-taking behaviour (Peeters et al., 2023). Recruitment into some recent trials was moreover negatively impacted by restrictions during the COVID-19 pandemic (Halvorsen et al., 2024; Peeters et al., 2024).

The review also identified that CAT methods are primarily based on mass spectrometry, with considerable variability in how the results are interpreted and used. For example,. there is no clear or accepted classification of adherence by CAT, complicating attempts to compare studies. Some studies consider a participant adherent only if there is 100% concordance between their prescribed and detected AHDs, and consider all other results to represent nonadherence, while others differentiate between categories such as “partial” and “complete” nonadherence, though the thresholds for these categories vary. Such discrepancies are a significant barrier to the development of a cumulative evidence base.

Historically, adherence of 80%, adapted from earlier studies based on pill counts and Medication Event Monitoring Systems or MEMS, has been accepted as an acceptable level of adherence, and correlates with cardiovascular outcomes (Valgimigli et al., 2019; Bansilal et al., 2016). Some of the studies in this review have applied this threshold to CAT. However, the validity of this approach with a point-in-time assay such as LC-MS/MS of serum or urine, is questionable. For example, a patient prescribed 4 AHDs who omits their diuretic on a day they have to travel to their hospital appointment, would have an adherence rate of 75% and be considered non-adherent. Labelling such a participant as “nonadherent” (as compared with “partially adherent”) may obscure the distinction between “perfect” and suboptimal adherence patterns and their causes and origins, and may impede the ability of clinicians to interpret these results. Indeed, this case example could represent a patient who is fully committed to their hypertension regimen and engaged with appropriate self-management. Omitting the diuretic dose in this instance can be classified as the kind of careful self-regulation that might be required to attend a clinical appointment, particularly for an older person with mobility limitations. Without some qualitative and contextual patient history the CAT result alone may provide a misleading clinical picture of how medicines are being used.

Few studies reported according to a theoretical framework. The minimum reporting criteria set out in the EMERGE guideline are not commonly adopted in clinical research on this topic. This guideline suggests that researchers define phases of adherence clearly including initiation (when the patient takes the first dose of a prescribed medication), implementation (the extent to which a patient’s actual dosing corresponds to the prescribed regimen), persistence (the length of time between initiation and the last dose) and discontinuation (the end of therapy, after a last dose is taken and no more doses are taken thereafter without a prescriber’s order) (De Geest et al., 2018). It is not clear whether authors are unaware of this guideline or choose not to refer to it for another reason. Recognising the potential for adherence to confound results in blood pressure trials, the Non-adherence Academic Research Consortium within the European Society of Cardiology have produced a consensus report providing a framework for reporting, interpreting and analysing medication non-adherence in cardiovascular clinical trials (Valgimigli et al., 2019). This is particularly relevant for trials of invasive and irreversible interventions such as RDN, and is reflected in the number of studies of RDN included in this review.

There remains considerable variation in terminology used in this topic. Articles published as recently as 2023 use the term “compliance” for medication adherence (Kustovs et al., 2023). A lack of standardised terminology may hinder effective literature searches, making it difficult to compare studies, aggregate data, and draw conclusions. Only one of the included papers used the term “chemical adherence testing” (Osula et al., 2022). Other terms used include biochemical adherence testing, therapeutic drug monitoring, drug screening, drug assays, drug measurement, compliance testing, and many others. The lack of consensus around terminology, definitions and methods may obscure the scope and findings of research, and is an added challenge to evidence synthesis in this area (Wunder et al., 2019).

There is some evidence that CAT itself improves adherence and BP control, regardless of the CAT result (Gupta et al., 2017b), however the quality of this supportive evidence is currently limited to observational evidence and some preliminary RCTs are beginning to appear (Halvorsen et al., 2024; Peeters et al., 2024; Morrissey et al., 2023). CAT may provide a useful impetus to consultations around medication adherence. When reported, communicating CAT results to patients was found to improve blood pressure control (Gupta et al., 2017b). Despite this, few of the included studies indicated that CAT results were communicated to patients or participants. While it is possible that such feedback occurred as part of clinical practice without being reported in the published research, the impact and optimum manner of such feedback is of crucial importance and requires further elucidation, given the concerns about the potential for CAT to negatively impact the patient-physician relationship (Schesing et al., 2020). Concerns have been raised about the ethicality of CAT, which is problematic if CAT is not introduced in a transparent and sensitive manner, with verbal informed consent (Lane et al., 2022).

Most studies measured adherence at a single point in time. This has valuable diagnostic utility if the clinician’s aim is to identify treatment-resistant hypertension, determine whether screening for secondary causes of hypertension is necessary, or to determine a patient’ suitability for specialised treatments such as RDN. However, the correlation between point-in-time CAT and longer-term medication adherence patterns remains unclear (Wunder et al., 2019). The potential need for ongoing chemical adherence monitoring, as part of an effort to optimise long-term management and cardiovascular risk reduction, must be considered. With this in mind, the demonstrated utility of CAT in diverse healthcare settings, including primary care and not just in specialised centres, is a welcome development, however appropriate cost-effectiveness evaluations are required to determine whether the resources required to implement CAT are justified.

### Limitations and methodological considerations

The strengths of this study include the broad and inclusive search strategy, the number of records reviewed and the rigorous screening and review process. However, we excluded the grey literature such as published abstracts without a full-text manuscript; this could have captured additional studies and may have provided evidence of novel approaches to CAT implementation in hypertension. Our included studies were limited to the English language. Initial screening of title and abstracts did not require decisions by two reviewers but all decisions in the full text screening and quality appraisal were confirmed by a pair of reviewers. Conflicts and uncertainties were resolved through discussion until consensus was reached. This team-based approach to evidence synthesis with reviewers and information retrieval specialists from diverse academic and clinical backgrounds helped to limit the biases that can affect evidence synthesis (Johnson and Hennessy, 2019).

## Conclusion

The current body of evidence demonstrates considerable variability in the approach to implementing CAT for hypertension management in clinical practice, and a paucity of randomised controlled trials to evaluate its impact. Future research could (i) adopt a cohesive theoretical framework including clear operational definitions to standardise the approach to this important topic; and (ii) further explore the impact of CAT on clinical outcomes using RCTs.
