# Supplementary material for: Chemical adherence testing in the clinical management of hypertension: a scoping review
Source: Front Pharmacol. 2024 Nov 6;15:1452464. doi: 10.3389/fphar.2024.1452464 (PMC11576289; doi:10.3389/fphar.2024.1452464)
Supplement: Supplementary file 1 [file Table1.docx]

Supplementary table 1 – Characteristics of CAT in included studies

| Study ID | Title | CAT method | CAT frequency | Participants informed in advance of CAT | Result fed back to participants/ patients | Adherence definition | Key Findings: Adherence |
| --- | --- | --- | --- | --- | --- | --- | --- |
| Peeters 2024 | Antihypertensive drug concentration measurement combined with personalized feedback in resistant hypertension: a randomized controlled trial | UHPLC-MS/MS of dried blood spot | Multiple | Yes | Yes | "The adherence data were converted into a binary variable by combining total nonadherent and partially nonadherent together." p.171 | At baseline: 70% adherence in intervention arm, 71% in SoC arm.After 12 months of follow-up: 93% adherence in intervention arm, 71% adherence in SoC arm. Recruitment stopped early due to futility. Physicians' estimations of adherence incorrect in 31% of patients (of which 36% consisted of nonadherence that was not recognised by the physician). Trial participation improved blood pressure in both study arms which sustained throughout a follow-up period of 12 months, but failed to establish any difference blood pressure and thereby resistant hypertension between the arms. |
| Kario 2023 | Anti-hypertensive medication adherence in the REQUIRE trial: post-hoc exploratory evaluation | LC-MS/MS of urine | Once | Not stated | Not stated | Adherence rates were classified into four categories: full adherence (100%), greater partial adherence (75–99%), lesser partial adherence (1–74%), and non-adherence (0%). | At baseline, 45% (26/58) patients showed poor adherence. |
| Kustovs 2023 | Opportunities of Amlodipine as a Potential Candidate in the Evaluation of Drug Compliance during Antihypertensive Therapy | UPLC of plasma for amlodipine only | Once | Not stated | Not stated | Not stated | Adherence rates not stated |
| Seleznev 2023 | Therapeutic Drug Monitoring in Arterial Hypertension | HPLC MS/MS of blood  (quantitative) | Once | Not stated | Not stated | Not stated | In patients with uncontrolled AH, the concentration of AHD was not less than in patients with controlled AH |
| Curneen 2023 | Major disparities in patient-reported adherence compared to objective assessment of adherence using mass spectrometry: A prospective study in a tertiary-referral hypertension clinic | LCMSMS spot urine | Once | No | Not stated | Subjects who demonstrated both self-reported and LC-MS/MS evidence of antihypertensive medication adherence of all reported antihypertensives were characterised as adherent, whilst patients who reported either nonadherence or adherence without LC-MS/MS evidence of adherence were classified as nonadherent | 27.4% of participants demonstrated true adherence to their self-reported antihypertensives, despite 75.3% (0.6-0.8) reporting adherence. There was no statistical significance between groups of objectively nonadherent and adherent individuals. |
| Peeters 2023 | Monitoring antihypertensive drug concentrations to determine nonadherence in hypertensive patients with or without a kidney transplant | HPLC-MS/MS of plasma | Once | Yes, but patients were not informed specifically when the measurement would take place. | Not stated | A patient was categorised as adherent if all expected drugs were detected, partially nonadherent when at least one of the expected AHDs was detected, and completely nonadherent when none of the prescribed AHDs was detected. | There was no difference in adherence rates between patients in whom blood was sampled on the day informed consent was signed, and patients in whom blood was sampled at least one day after signing informed consent.  The overall proportion of patients adherent to AHDs was 78.2% (n = 111 patients). From the nonadherent patients, 87.8% was partially nonadherent and 12.2% non-adherent for all measured AHDs. Patients with a kidney transplant were more likely to be adherent to AHDs.Patients with resistant hypertension were less likely to be adherent to AHDs than patients not having resistant hypertension |
| Osman 2023 | An Innovative Chemical Adherence Test Demonstrates Very High Rates of Nonadherence to Oral Cardio-Metabolic Medications | LC-MS/MS of urine | Once | Yes, immediately before obtaining urine sample | No | Patients were determined to be nonadherent if at least 1 of their prescribed cardio-metabolic medication was not detected in their urine sample | 45% of the cohort was nonadherent to at least 1 of the prescribed cardio-metabolic medications with 14.2% of the cohort found to be nonadherent to all prescribed cardio-metabolic medication  Higher number of prescribed medications and increased age were significant predictors of nonadherence. |
| Georges 2022 | Psychological determinants of drug adherence and severity of hypertension in patients with apparently treatment-resistant vs. controlled hypertension | LC-MS/MS of urine. | Once | Urines were collected on the day of the signature of the written informed consent | Not stated | Full drug adherence, partial drug adherence and total non-adherence were defined as the presence of all, part or none of the prescribed drugs in the urine, respectively. Drug adherence was defined as the percentage of prescribed antihypertensive drugs which were effectively detected in the urine. | Mean drug adherence was significantly lower in patients with aTRH. In patients with aTRH, independent predictors of poor drug adherence were somatisation, smoking and low acceptance level of difficult situations, accounting for 41% of the variability in drug adherence. |
| Sheppard 2022 | Measuring adherence to antihypertensive medication using an objective test in older adults attending primary care: cross-sectional study. | LC-MS/MS urine | Once | No | Not stated | Medication adherence was defined as a binary outcome; adherent patients were those in whom all prescribed antihypertensive medications were present in their urine sample. Non-adherent patients were those in whom only some or none of their prescribed medications were detected in the urine sample. | A total of 27/191 participants (14.2%) reported not taking all of their medications on the day of urine sample collection. However, LC-MS/MS analysis of samples revealed only 4/27 (9/191 in total; 4.7%) were non-adherent to some of their medications. patients prescribed more antihypertensive medications (OR 0.24, 95%CI 0.09 to 0.65) were less likely to be adherent to antihypertensive medication. No other factors predicted non-adherence to antihypertensive medication |
| Groenland 2022 | Clinical characteristics do not reliably identify non-adherence in patients with uncontrolled hypertension | LC-MS/MS urine | Once | No. Verbal consent for CAT on day of clinical appointment. | Not stated | Classified as adherent (all of the prescribed medications detected) or non-adherent (atleast one of the prescribed medications not detected). Non-adherence was further categorised into full non-adherence (complete absence of any prescribed antihypertensive medications in the blood or urine sample) and partial non-adherence (presence of fewer medications than prescribed in blood or urine sample) | Prevalence of non-adherence to antihypertensive drugs was 19% in the UMCU and 44% in the Heartlands Hospital population. It is not possible to sufficiently accurately predict whether a patient will be adherent with antihyperten- sive treatment based on a combination of either clinical characteristics or self-reported barriers to medication adherence. This emphasises the need for direct and objective chemical adherence testing in routine clinical practice. |
| Peeters 2022 | Introducing the importance and difficulties of a threestep approach to improve nonadherence to antihypertensive drugs: A case series | IPLC-MS/MS of dried blood spot | Multiple | Yes | Yes | Narrative description (case series) | The importance of a three-step approach to improve drug therapy in patients with resistant hypertension including identification of nonadherence, determination of the underlying cause and finding a personalized solution. |
| Osula 2022 | Comparison of Pharmacy Refill Data With Chemical Adherence Testing in Assessing Medication Nonadherence in a Safety Net Hospital Setting | plasma LC-MS/MS. Also performed CAT of statins. | Once | Yes | Not stated | If 1 or more of the prescribed cardiovascular drugs was below detection limit, the patient was considered nonadherent by CAT | 17% were nonadherent to at least 1 antihypertensive drug by CAT. There were no significant differences in age, sex, race or ethnicity, presence of comorbidities, or enrollment in the financial assistance program between adherent and nonadherent patients.  Pharmacy refill data have modest sensitivity and specificity and low positive predictive value in detecting medication nonadherence when compared with CAT from serum samples. The positive predictive values for pharmacy refill data detecting nonadherence were very low for all antihypertensives (11%-27%). |
| Wang 2021 | Monitoring Antihypertensive Medication Adherence by Liquid Chromatography-Tandem Mass Spectrometry: Method Establishment and Clinical Application. | LC-MS/MS urine (quantitative) | Once | Not stated | Not stated | Not stated | The results revealed that antihypertensive drugs detected in urine samples from 92% subjects were consistent with their prescriptions during the 28-day run-in periods. |
| Buffolo 2021 | Assessment of Anti-Hypertensive Drug Adherence by Serial Aldosterone-To-Renin Ratio Measurement | LC-MS/MS of urine and plasma; serial aldosterone-to-renin (ARR) measurement |  | Not stated | Not stated | ΔARR threshold of 48% | After 2 and 8 weeks, assumption of ARBs or ACEi was confirmed by TDM in all patients of the cohort treated with RAAS inhibitors. Delta ARR (ΔARR), defined as relative change in ARR before and after treatment initiation, provided high accuracy for determination of therapeutic compliance, with an AUC of 0.900 at 2weeks and 0.886 at 8weeks. A cut-off of −48% of ΔARR provided 90% sensitivity and 75% specificity, at 2 and 8weeks. ΔARR is a powerful test, cheap and widely available, to accurately identify the non-adherence to RAAS inhibitors treatment. |
| Beernink 2021 | Biochemical Urine Testing of Medication Adherence and Its Association With Clinical Markers in an Outpatient Population of Type 2 Diabetes Patients: Analysis in the DIAbetes and LifEstyle Cohort Twente (DIALECT) | LC-MS/MS of urine | Once | No | Not stated | Patients were considered adherent if all screened medications were detected in the urine and nonadherent if at least one of the screened medications was not detected. | Adherence rates to AHDs were 92%. Higher BMI, current smoking elevated serum LDL cholesterol, high HbA1c, presence of DKD, and presence of macrovascular disease were significantly associated with nonadherence. |
| Schafer 2021 | Eligibility for Baroreflex Activation Therapy and medication adherence in patients with apparently resistant hypertension | Gas chromatography-mass spectrometry (GC-MS) of urine | Once | Not stated | Not stated | Mean adherence was defined as the ratio of detected to traceable antihypertensive drugs. Complete adherence was defined as 100% proof of detectable medication, partial adherence as ≥66 but <100%, partial non-adherence as <66 but >0%, and complete non-adherence was defined as no detection of any traceable antihypertensive medication. | 78.7% of patients showed adherence to ≥66% of the prescribed drug regimes,  including 54.7% with complete adherence. 21.3% patients showed partial or complete non-adherence. |
| Lauder 2021 | Drug adherence and psychosocial characteristics of patients presenting with hypertensive urgency at the emergency department | LC-HRMS/MS of blood and urine | Once | Not stated | Not stated | Patients were classified nonadherent if less than 80% of the prescribed antihypertensive drugs were detectable in urine or plasma using LC-MS/MS. If at least 80% of the prescribed AHDs were detectable, the patient was classified as adherent. | 24% were nonadherent to their antihypertensive medication.  Male sex, higher numbers of antihypertensive drugs, and treatment with diuretics and/or calcium channel blockers were associated with nonadherence. There was no association between health literacy, depression or anxiety scores and nonadherence. |
| Wunder 2019 | Adherence to antihypertensive drug treatment in patients with apparently treatment-resistant hypertension in the INSPiRED pilot study. | LC-MS/MS urine | Multiple | The informed consent signed by patients enrolled in the trial stated that a urine sample would be collected at every visit in order to check the presence of antihypertensive drugs in the urine. This was not reminded on subsequent visits. | Not stated | Adherence rate (percentage of number of detected vs. prescribed medical drugs) reported. | At baseline, median adherence ranged from 0 to 100% with a median of 73.2%. In further urine samples collected during the following up to 17 months every individual patient exhibited considerable changes in the adherence rate, neither a constancy nor a tendency could be deduced. |
| Pelouch 2019 | The Assessment of Serum Drug Levels to Diagnose Non-Adherence in Stable Chronic Heart Failure Patients | *Included only drugs the patient confirmed as having taken the evening before and the morning of the visit. LC-MS/MS of serum.* | Multiple | No | Not stated | Patients in whom the serum level of at least one drug was below the limit of detection was labelled as non-adherent. | All of evaluated drugs were detected in the serum of 61 patients (75%) and the criteria for non-adherence were  fulfilled in the remaining 20 patients (25%). One of all drugs was undetectable in 10 patients (12.5%), more than  one in the serum of 9 patients (11%). None of the evaluated drugs was detectable in 1 patient (1.5%). |
| Hayes 2019 | Measuring adherence to therapy in apparent treatment-resistant hypertension: A feasibility study in Irish primary care | LC-MS/MS of urine | Once | Patients were informed by letter  that the urine assessment was to examine "the success of your tablets in controlling  your blood pressure" | Not stated | Patients were described as being fully or partially adherent, or fully non-adherent depending on the ratio of the number of drugs present in their urine divided by the number of drugs prescribed. | 174 patients were fully adherent to treatment (74%), 56 partially adherent (24%), and five fully non-adherent (2%). |
| deJager 2018 | Medication adherence in patients with apparent resistant hypertension: findings from the SYMPATHY trial | LC-MS/MS of serum. | Multiple | No | Not stated | Medication adherence was documented in three different categories: adherent (81%-100% match prescribed vs. measured), poorly adherent (1-80% match prescribed vs. measured) and completely nonadherent (0% match prescribed vs. measured). | 68% of the patients was either completely nonadherent or poorly adherent. 32% were fully adherent.  Adherence at baseline declined significantly with the increase of number of prescribed drugs. : for every one pill more prescribed, 0.785 prescribed pill was less detected in blood (B = 0.785, P < 0.001). |
| Sandbaumhuter 2018 | Medication adherence during laboratory workup for primary aldosteronism: Pilot study | LC-MS/MS plasma | Multiple | No | Not stated | Nonadherence was defined as a plasma drug result not consistent with prescriptions. Nonadherence due to missing drug intake (NMI) corresponded to a negative plasma result despite a prescription. Partial NMI was defined, if one or more of prescribed and tested drugs were absent in plasma and complete NMI, if all tests were negative. | Nonadherence was detected in 33% of patients on the first visit, in 25% on the second visit, and in 46% for both visits  combined. |
| Sutherland 2018 | Assessment of Patient Medication Adherence, Medical Record Accuracy, and Medication Blood Concentrations for Prescription and Over-the-Counter Medications | LC-MS/MS of plasma | Once | Not stated | Not stated | Subset of oral medications with a half-life greater than 4 hours or an empirical detection rate of 70% or more tested. The resulting subset of 189 detected drugs was used to calculate the percentage of all prescribed medications to which a patient was adherent. | (1) medications are frequently detected despite lack of prescription evidence in the EHR, (2) patient adherence varies by health care setting, (3) being adherent to 1 medication is a positive determinant of adherence to other medications, and (4) drug-drug interactions were common and occurred more frequently among patients with medications that were Detected, not prescribed (DNP). Using this measure, the median adherence rate was 78% (IQR, 57%-100%) in the ED care cohort |
| Avataneo 2018 | Therapeutic drug monitoring-guided definition of adherence profiles in resistant hypertension and identification of predictors of poor adherence | LC-MS/MS of plasma | Once | Yes; informed at short notice to minimise white coat adherence. | Not stated | Fully adherent patients had detectable plasma concentrations of all prescribed drugs, partially adherent patients showed detectable concentrations of only a part of all prescribed drugs, totally nonadherent patients had undetectable concentrations of all the prescribed drugs. | 24% of patients partially complied to treatment and 18% were nonadherent. A statistically significant concordance (P = 0.002) was observed between specialist opinion and TDM results, while no significant association was found between questionnaire and TDM results. All the TDM-defined non-adherent patients self defined as adherent in the questionnaire. Nonadherence was associated with high diastolic blood pressure, high heart rate, previous onset of stroke and previous use of invasive treatments, including renal denervation or baroreceptor stimulation. |
| Petit 2018 | Impact of psychological profile on drug adherence and drug resistance in patients with apparently treatment-resistant hypertension | LC-MS/MS of urine | Once | Urines were collected on the day of signature of the written informed consent | Not stated | Drug adherence was defined as the percentage of prescribed antihypertensive drugs which were effectively found in the urine. | The proportion of adherent, partly adherent and totally non-adherent patients was 29, 40 and 31%, respectively. In regression analysis,  independent predictors of poor drug adherence were recent hospital admission for hypertension, a lower ability to put things into perspective when facing negative events and a higher tendency to somatize, accounting for 51% of variability in drug adherence. Independent predictors of treatment resistance were a higher recourse to the strategies of blaming others and oneself, accounting for 37% of variability in drug treatment resistance. The adherence level was positively correlated with higher education level. On the contrary, adherence was negatively associated with total number of drugs/day, being a woman and having been admitted to the hospital for hypertension in the last year. |
| Gupta 2017 (1) | Risk Factors for Nonadherence to Antihypertensive Treatment | urine LC-MS/MS (UK); Serum LC-MS/MS (Czech Rep) | Once | Yes, on the day of analysis. |  | The response variable was a binary indicator of medication class-specific nonadherence defined as absence of at least 1 medication from the associated class in the HPLC-MS/MS-based test results. Thereafter, participants classed as "partial nonadherence", "total nonadherence" or "total adherence". | UK: the rates of any, partial, and total nonadherence were 41.6%, 27.1%, and 14.5%, respectively. The risk of nonadherence increases with the number of prescribed BP-lowering medications. This association was independent of age, sex, as well as the class of prescribed antihypertensive medications. Czech Rep: The prevalence of any, partial, and total nonadherence was 31.5%, 19.5%, and 12.1%, respectively. On average, every increase in the number of prescribed antihypertensive medications was associated with 85% and 77% increase in the odds of the nonadherence in the UK and Czech populations, respectively |
| Jones 2017 | Therapeutic drug monitoring of amlodipine and the Z-FHL/HHL ratio : adherence tools in patients referred for apparent treatment-resistant hypertension | LC-MS/MS of plasma for amlodipine (quantitative assay), Z-FHL/HHL (z-phenylalanine-histidine-leucine/hippuryl-histidine-leucine) ratio. for ACE inhibitor. | Multiple | Not stated | Not stated | For enalapril: Z-FHL/HHL ratio of <1.75. For amlodipine: Amlodipine concentrations <2.5 ng/mL deemed to be undetectable. Indeterminate, 2.5 - 7 ng/mL and Steady state >7ng/mL | 20% of patients had an undetectable amlodipine concentration and 27% had unsuppressed ACE, and this was associated with significantly higher BPs compared with those with steady-state amlodipine concentrations or suppressed ACE. |
| Hamdidouche 2017 | Routine urinary detection of antihypertensive drugs for systematic evaluation of adherence to treatment in hypertensive patients | LC-MS/MS of urine. Adherence to ACEis was determined from spot urine AcSDKP/creatinine ratio | Multiple | No | No | Full nonadherence was defined as complete absence of any prescribed antihypertensive medications or their metabolites when appropriate. Partial nonadherence was defined as the absence of at least one, but not all, prescribed antihypertensive drugs. Full adherence was defined by the presence of all prescribed antihypertensive drugs. | The prevalence of directly measured nonadherence (either full or partial) by urine drug detection was approximately 10%. Compared with adherent patients, those who did not adhere to their treatment had a higher number of antihypertensive pills and drugs, cotreatment with cardiovascular drugs, and total concurrent medications and pills. After adjustment for age, home and office BP were higher in the nonadherent than the adherent group. All other clinical characteristics, such as age, sex, hypertension duration, BMI, smoking, and type II diabetes, did not differ between adherent and nonadherent patients There was no significant association between four-item Morisky Medication Adherence Scale score and directly measured nonadherence. |
| Gupta 2017 (2) | Biochemical Screening for Nonadherence Is Associated With Blood Pressure Reduction and Improvement in Adherence | UK participants: LC-MS/MS of urine  Czhech participants: LC-MS/MS of serum | Multiple | UK: verbal consent once informed about the purpose of urine collection on day of clinical appointment.   Czechia: written consent. serum samples collected on initial visit as part of routine service. | Yes | Patients whose baseline urine analysis by LC-MS/MS did not detect at least one of the prescribed antihypertensive medications were classified as initially nonadherent. Those whose baseline urine analysis detected all prescribed antihypertensive medications were classified as initially adherent.  Czech Rep.: Patients whose baseline serum analysis by LC-MS/MS did not detect at least one of the prescribed antihypertensive medications were classified as nonadherent. | Of 238 UK patients included in this analysis, 165 and 73 were classified as initially adherent and nonadherent (respectively) based on the results of their first LC-MS/MS urine test. CAT is associated with better adherence to antihypertensive treatment and improved BP control in initially nonadherent patients. Initially nonadherent patients who become adherent reach BP similar to that of persistently adherent patients. |
| Kocianova 2017 | Heart rate is a useful marker of adherence to beta-blocker treatment in hypertension. | UHPLC-MS of serum (quantitative) | Multiple | No | Not stated | A patient was classified as adherent at the particular measurement if the serum level was within therapeutic range. A patient was classified as non-adherent if the serum level was below the therapeutic range or undetectable. | Only 55.4% of betablocker serum level measurements were within the therapeutic range, thus we classified these patients as adherent and the remaining 44.6% as non-adherent. Heart rate above 75.5 beats per minute predicted non-adherence to beta-blocker treat ment with a sensitivity of 62.5%, specificity 86.8% and AUC ROC 0.802 (p<.001) |
| McNaughton 2017 | Systolic Blood Pressure and Biochemical Assessment of Adherence: A Cross-Sectional Analysis in the Emergency Department | LC-MS/MS of serum | Once | No | Not stated | The ratio of the number of antihypertensives detected in blood to the number of antihypertensives that had been prescribed (of the 14 assay antihypertensives) was used to categorize patients as adherent (ratio=1.0) or nonadherent (ratio<1.0). | By the liquid chromatography mass spectrometry assay, nearly 28% of patients were nonadherent to prescribed antihypertensive medications.79.0% of patients prescribed <3 antihypertensives were adherent, and 58.8% of patients prescribed ≥3 antihypertensives were adherent. 32% complete nonadherence among the 202 patients who reported being adherent by the self-report n this study. Antihypertensive nonadherence wasassociated with higher BP in the ED among patients who had a primary care provider and health insurance and who were prescribed ≥3 antihypertensives. |
| Bohlender 2017 | Medication adherence during work-up for Conn syndrome | LC-MS/MS of plasma (quantitative) | Multiple | No | Not stated | Nonadherence was defined as a plasma drug result not consistent with prescriptions. Nonadherence due to missing drug intake (NMI) corresponded to a negative plasma result despite a prescription. Partial NMI was defined, if one or more of prescribed and tested drugs were absent in plasma and complete NMI, if all tests were negative. | Nonadherence was detected in 33% of patients on the first visit, in 25% on the second visit, and in 46% for both visits combined. Using indexed plasma drug concentrations and expected Cmin as a threshold for same-day drug intake, we additionally revealed up to four nonadherent patients compared with only qualitative plasma drug screening. |
| Schmieder 2016 | Adherence to antihypertensive medication in treatment-resistant hypertension undergoing renal denervation | LC-MS/MS of urine | Once | No | Not stated | Adherence data were categorized as complete adherence, indicating that all prescribed antihypertensive drugs were detected; partial adherence, indicating that, at maximum, 1 of the prescribed drugs was missing; and nonadherence, indicating that ≥2 of the prescribed drugs were not found in the toxicological analysis (numeric adherence). In a second set of analyses, we defined adherence as detection of ≥80% of the detectable drugs and nonadherence as detection of <80% measured in the urine analysis. These criteria are established for the judgment of adherence in pharmacological studies. | Actual intake of all antihypertensive drugs was detected at baseline and at 6 months after renal denervation in 56% and 66% patients, respectively. |
| Patel 2016 | Screening for non-adherence to antihypertensive treatment as a part of the diagnostic pathway to renal denervation | HPLC-MS/MS of urine | Once | Verbal consent from patients on day of clinic visit | Not stated | *Not explicitly stated*. "Partial" and "total" nonadherence described in results. | 33% of patients who underwent CAT (and 23.5% of those referred for renal denervation) were biochemically non-adherent to antihyperten- sive treatment. |
| Beaussier 2015 | True antihypertensive efficacy of sequential nephron blockade in patients with resistant hypertension and confirmed medication adherence | 1. plasma irbesartan concentration using HPLC. 2. urine AcSDKP/ creatinine ratio for ACEi. multiple occasions. Combined with self-report and pill count. | Once | Yes | Not stated | 1 point allocated to each of (1)HPLC of irbesartan, (2)urine AcSDKP/creatinine ratio as marker of ACEi adherence, (3) pill counting (80%), and (4) participant self-report.  On the basis of the score distribution, a score below 2 was defined as low medication adherence and above 2 as acceptable medication adherence | 134 (81.7%) had acceptable medication adherence and 30 (18.3%) had low medication adherence |
| Ewen 2015 | Blood pressure reductions following catheter-based renal denervation are not related to improvements in adherence to antihypertensive drugs measured by urine/plasma toxicological analysis | LC-MS/MS of plasma and urine | Multiple | "in order to prevent stimulation of white-coat adherence, patients were not informed about the timing and execution of the measurement" (p.8) | Not stated | Adherence was defined as 100 % confirmation of all prescribed antihypertensive drugs by LCHR-MS/MS analysis. Patients in whom the analysis confirmed the presence of less medication than prescribed (antihypertensive drug intake detected\100 %) were classified as non-adherent. | At baseline, complete adherence to all prescribed antihypertensive agents was observed in 52% patients, 46% patients were partially adherent, and 2% patients were completely non-adherent.  There were no significant differences between adherent (52 %) and non-adherent (48 %) patients at baseline with respect to age, sex, body mass index, office and ambulatory BP, office heart rate, coronary artery disease, hypercholesterolemia, type 2 diabetes, cystatin c GFR, number of all prescribed drugs and number of antihypertensive drugs. Mean adherence to prescribed treatment was significantly reduced from 85.0 % at baseline to 80.7 %, 6 months after renal denervation. |
| Florczak 2015 | Assessment of adherence to treatment in patients with resistant hypertension using toxicological serum analysis | LC-MS/MS of serum | Once | Not stated. | Not stated | Nonadherence defined as at least 1 of the prescribed medications below the LOQ. Where none of the prescribed drugs could be detected, patients were considered completely nonadherent. Where at least 1 (but not all) of the prescribed drugs could not be detected, patients were consid ered as partially nonadherent. | Nonadherence criteria were met in 86.1% of patients. In 13.9% of patients completely nonadherent and 72.2% partially nonadherent. |
| Velasco 2015 | Cost-Effectiveness of Therapeutic Drug Monitoring in Diagnosing Primary Aldosteronism in Patients With Resistant Hypertension | LC-MS/MS; HPLC; GC; Spectrofluorometry | Once | Not stated | Not stated | Patients with serum levels of one or more prescribed antihypertensive medications below the minimal detection limit were considered to be nonadherent. Medication nonadherence ratio was calculated as the number of undetectable antihypertensive medications divided by the total number of antihypertensive medications tested. | Of the 78 patients tested, 43 (55%) were shown to be nonadherent to at least one medication prescribed despite self-reported adherence. prevalence of PA in adherent patients with TRH by TDM was significantly higher than in nonadherent patients (28% vs 8%, P<.05). Screening for PA using a TDM-guided approach was cost-saving compared with routine unselective screening. |
| Tomaszewski 2014 | High rates of non-adherence to antihypertensive treatment revealed by high-performance liquid chromatography-tandem mass spectrometry (HP LC-MS/MS) urine analysis | LC-MS/MS of urine. | Once | Yes (timing not stated). "Prior to screening, the patients were advised that their urine sample would be assayed for presence of BP lowering drugs" | Yes | Total non-adherence to antihypertensive treatment was defined as complete absence of any prescribed antihypertensive medications (or their metabolites where appropriate) in a spot urine sample on screening. Patients whose urine analysis confirmed the presence of fewer medications than prescribed were classified as partially non-adherent. | Total non-adherence 10.1%, partial non-adherence 14.9%. the highest percentage of total non-adherence with antihypertensive treatment was among those referred for renal denervation (23.5%). |
| Rosa 2014 | Importance of thorough investigation of resistant hypertension before renal denervation: should compliance to treatment be evaluated systematically?. | LC-MS/MS of serum | Once | No | Not stated | To be considered as compliant to the treatment, all studied drugs needed to be positively tested. | Among 72 patients with essential hypertension, non-compliance was confirmed in 27 patients (13.2% of the original cohort; partial non-compliance was present in 10 patients, full in 17 patients). This means, only 45 patients (21.9%) were really true-resistant hypertensive |
| Brinker 2014 | Therapeutic drug monitoring facilitates blood pressure control in resistant hypertension | LC-MS/MS; HPLC; GC; Spectrofluorometry | Once | Not stated | Yes | Subjects with serum levels of at least 1 prescribed antihypertensive drug below the minimal detection limit were considered to be nonadherent. | 54% of patients who underwent TDM were found to be nonadherent to treatment. 32% had undetectable levels of all drugs, 22% had at least 1 undetectable drug. All nonadherent patients initially denied missing any doses of their antihypertensive medications in the 24 h before TDM.  When the 16 patients in the nonadherent group were provided with TDM results, 2 attributed their nonadherence to memory loss, 3 described debilitating fatigue not previously reported during the first encounter, and 5 reported drug cost as a major barrier to adherence. |
| Jung 2013 | Resistant hypertension? Assessment of adherence by toxicological urine analysis | LC-MS/MS of urine (lercanidipine and nitrates not included) | Once | No | Yes | Adherence defined as all AHDs prescribed detected in urine. Non adherence included 'complete nonadherence' (none of the prescribed drugs detected) or 'incomplete adherence' (percentage of prescribed drugs detected reported for these patients). | 52.6% non-adherent (partial or full). Rates of non-adherence comparable among drug classes. |
| Strauch 2013 | Precise assessment of noncompliance with the antihypertensive therapy in patients with resistant hypertension using toxicological serum analysis | LC-MS/MS blood | Once | No | Not stated | Total noncompliance was defined as the absence of all measured antihypertensive drugs. Partial noncompliance was calculated as the absence of serum levels of at least one, but not all AHDs apparently taken. | 23% partially noncompliant and 24% totally noncompliant. Lower compliance was found in less educated patients in comparison with higher educated patients. No correlation was found between the number of prescribed AHDs and compliance |
| Ceral 2011 | Difficult-to-control arterial hypertension or uncooperative patients the assessment of serum antihypertensive drug levels to differentiate non-responsiveness from non-adherence to recommended therapy | LC-MS/MS of serum | Once | Not stated | Not stated | Patients in whom the serum level of at least one drug was below the limit of quantification were labeled as non-adherent. | 34.5% patients were deemed compliant . None of the evaluated drugs were detectable in 34.5% of the study group.  In 8.3% patients, we identified AHDs that were neither prescribed nor reported to be used by the patients. |
| Azizi 2006 | Assessment of Patients' and Physicians' Compliance to an ACE Inhibitor Treatment Based on Urinary N-Acetyl Ser-Asp-Lys-Pro Determination in the Noninsulin-Dependent Diabetes, Hypertension, Microalbuminuria, Proteinuria, Cardiovascular Events, and Ramipril | Measurement of urinary N-acetyl_Ser-Asp-Lys-Pro (endogenous biomarker of ACE inhibition) | Once | Not stated | Not stated | We defined truly adherent ramipril patients as those with urinary AcSKDP-to-creatinine ratio >4 nmol/mmol and truly adherent placebo patients as those with a ratio <4 nmol/mmol. | In the ramipril group, 35.3% of the patients had urinary AcSKDP-to-creatinine ratios <4 nmol/mmol in intention-to treat analysis, within the range of values indicating an absence of ACE inhibitor intake.   In the per-protocol analysis, 27.3% of the patients had urinary AcSKDP-to-creatinine ratios <4 nmol/mmol, indicating a lack of adherence to ramipril treatment   In the placebo group, 9.7% had AcSDKP-to-creatinine ratios <4 nmol/mmol, in the absence of any ACE inhibitor prescription explicitly mentioned in their case report form. |
|  |  |  |  |  |  |  |  |
| vanSchoonhoven 2018 | Cost-Utility of an Objective Biochemical Measure to Improve Adherence to Antihypertensive Treatment | N/A |  |  |  |  | Lifetime cost-utility was assessed from a UK healthcare payer perspective, using a Markov model.   Per patient, screening resulted in 0.020 incremental quality-adjusted life-years and a negative incremental cost of Â£495,  suggesting the intervention to be dominant compared with care as usual. Targeting younger patients or patients with apparent  resistant hypertension would further improve these outcomes. Modeling suggested that screening prevented 518 myocardial  infarctions and 305 stroke events in a cohort of 10000 male hypertensive patients. Using liquid chromatography-tandem  mass spectrometry-based biochemical analyses to improve adherence in hypertensive patients is likely to be an effective  and cost-saving strategy, especially in patients with apparent resistant hypertension |
