# Supplementary material for: Chemical adherence testing in the clinical management of hypertension: a scoping review
Source: Front Pharmacol. 2024 Nov 6;15:1452464. doi: 10.3389/fphar.2024.1452464 (PMC11576289; doi:10.3389/fphar.2024.1452464)
Supplement: Supplementary file 2 [file Table2.docx]

Supplementary Table 2 – Characteristics of study participants

| **Study ID** | **Title** | **N** | **Comparator group** | **Inclusion criteria** | **Exclusion criteria** |
| --- | --- | --- | --- | --- | --- |
| Peeters 2024 | Antihypertensive drug concentration measurement combined with personalized feedback in resistant hypertension: a randomized controlled trial | 100 | Intervention (communication tool) +SoC vs SoC only | ≥3AHDs including a diuretic or ≥4AHDs and Office/ABPM of at least 140 and/or 90mmHg. | eGFR <15ml/min, possible secondary causes of hypertension, unwillingness to do a 24h ABPM, insufficient understanding of language to read the PIL. |
| Kario 2023 | Anti-hypertensive medication adherence in the REQUIRE trial: post-hoc exploratory evaluation | 58 | None | Participants enrolled in RCT comparing ultrasound Renal Denervation vs sham procedure |  |
| Kustovs 2023 | Opportunities of Amlodipine as a Potential Candidate in the Evaluation of Drug Compliance during Antihypertensive Therapy | 81 | None | Subject age >18y, diagnosis of primary or secondary hypertension, taking amlodipine for at least six months | The exclusion criteria was severe hepatic impairment |
| Seleznev 2023 | Therapeutic Drug Monitoring in Arterial Hypertension | 46 | controlled vs uncontrolled BP considered as two groups | Aged >18y; established diagnosis of hypertension; compliance with recommendations on lifestyle modification; regular administration of any two AHDs in comination with indapamide for a month. | Patients connection with the organization or conducting of the study; Pregnancy. |
| Curneen 2023 | Major disparities in patient-reported adherence compared to objective assessment of adherence using mass spectrometry: A prospective study in a tertiary-referral hypertension clinic | 73 | None | Patients aged ≥18 years old on one or more antihypertensive medications who were attending the tertiary hypertension clinic were eligible for inclusion and were recruited at a routine clinic visit without prior knowledge of the study. |  |
| Peeters 2023 | Monitoring antihypertensive drug concentrations to determine nonadherence in hypertensive patients with or without a kidney transplant | 142 | Compared those who had received kidney transplant with those who had not. | Patients were recruited from the vascular outpatient clinic for hypertension and the nephrology outpatient clinic including patients with a kidney transplantation. ≥18 years or older, office BP at least 140 and/orat least 90 mmHg and were treated with at least two AHDsfor which an ultra-high-performance liquid chromatography-tandem mass spectrometry (UPLC-MS/MS) method was developed and validated. | Patients were excluded from participation if they were not able or willing to give informed consent or had an estimated eGFR less than 15 ml/min per 1.73 m2  patients were not included if they visited the outpatient clinic for the first time or if secondary causes of hypertension were expected but not confirmed yet |
| Osman 2023 | An Innovative Chemical Adherence Test Demonstrates Very High Rates of Nonadherence to Oral Cardio-Metabolic Medications | 106 | None | Patients attending renal clinic prescribed at least 1 oral cardiometabolic medication. | not stated |
| Georges 2022 | Psychological determinants of drug adherence and severity of hypertension in patients with apparently treatment-resistant vs. controlled hypertension | 144 | Controlled hypertensive patients | patients with aTRH confirmed with ABPM | age <18 years, estimated glomerular filtration (eGFR) <30 ml/min/1.73 m2 according to the CKD-EPI formula, body mass index 40 Kg/m2 and a history of myocardial infarction, stroke or polyvascular disease |
| Sheppard 2022 | Measuring adherence to antihypertensive medication using an objective test in older adults attending primary care: cross-sectional study. | 191 | None | Consecutive patients aged >65 years with an electronic medical record coded diagnosis of hypertension and prescribed at least one blood pressure lowering medication. Participating general practices were in areas of relatively low deprivation. |  |
| Groenland 2022 | Clinical characteristics do not reliably identify non-adherence in patients with uncontrolled hypertension | derivation group - 495 and validation group - 240 | None | NL: if BP targets were not met despite BP lowering treatment and/or suffered from target-organ damage, who were prescribed at least one AHD.  UK:Patients underwent biochemical drug screening in urine when either medication non-adherence was suspected by the treating hypertension specialist or when patients fulfilled the criteria for apparent resistant hypertension (in spite of concurrent use of three antihypertensive agents of different classes) |  |
| Peeters 2022 | Introducing the importance and difficulties of a threestep approach to improve nonadherence to antihypertensive drugs: A case series | 3 | None | 3 participants in RHYME-RCT (Peeters et al 2024) |  |
| Osula 2022 | Comparison of Pharmacy Refill Data With Chemical Adherence Testing in Assessing Medication Nonadherence in a Safety Net Hospital Setting | 77 | None | Uncontrolled hypertension (defined as office systolic BP ≥130mmHg and/or diastolic BP of ≥80mmHg.) | (1) presence of hypertensive emergency;  (2) history of active substance abuse such as alco- hol, cocaine, or narcotics; (3) uncontrolled psychiatric  disorder such as schizophrenia or major depression  based on diagnosis entered in electronic health record;  (4) pregnancy; (5) homelessness; (6) stage V chronic  kidney disease or end-stage renal disease (glomeru- lar filtration rate <15mL/min/1.73m2); (7) self-report of  nonadherence or unwillingness to follow medication  regimen prescribed by the primary care physicians  for any reason; (8) presence of white coat hyperten- sion (defined as normal home systolic BP of <130  and diastolic BP of <80mmHg and clinic systolic BP  ≥130mmHg or diastolic ≥80mmHg); and (9) inabil- ity to read or write English. |
| Wang 2021 | Monitoring Antihypertensive Medication Adherence by Liquid Chromatography-Tandem Mass Spectrometry: Method Establishment and Clinical Application. | 92 | None | Consecutive patients undergoing trial for catheter based trial to treat hypertension. (during 28 day run-in period) | not stated |
| Buffolo 2021 | Assessment of Anti-Hypertensive Drug Adherence by Serial Aldosterone-To-Renin Ratio Measurement | 40 + 40 | Healthy controls | Patients referred to hypertension unit with arterial hypertension with diagnosis of arterial hypertension according with ESC/ESH guideline aged between 18 and 80 years old | beta blockade, clonidine, diuretics, RAAS inhibitors, secondary hypertension  excl criteria for control group: Exclusion criteria for controls were 1) antihypertensive treatment both at recruitment time and/or during follow-up and 2) chronic treatment with non-steroidal anti- inflammatory drugs and contraceptive pill |
| Beernink 2021 | Biochemical Urine Testing of Medication Adherence and Its Association With Clinical Markers in an Outpatient Population of Type 2 Diabetes Patients: Analysis in the DIAbetes and LifEstyle Cohort Twente (DIALECT) | 457 | None | Patients with type 2 diabetes treat ed in the specialist outpatient clinic as part of routine secondary care |  |
| SchÃ¤fer 2021 | Eligibility for Baroreflex Activation Therapy and medication adherence in patients with apparently resistant hypertension | 75 underwent CAT | None | Patients with apparently resistant hypertension who attended the certified hypertension clinic. Patient undergoing screening for suitability for BAT (screening for white coat hypertension, secondary htn; optimisation of meds). *Unclear how they chose on whom to do CAT*: "In case of suspected non-adherence, selective adherence tests were performed." (75 of 345) |  |
| Lauder 2021 | Drug adherence and psychosocial characteristics of patients presenting with hypertensive urgency at the emergency department | 104 | None | Patients with previously diagnosed hypertension presenting with hypertensive urgencies (SBP 180 mmHg and/or DBP 110mmHg) | Pregnant women and patients presenting with hypertensive emergencies, defined as situations where high BP values are associated with acute hypertension-mediated organ damage were excluded |
| Schesing 2020 | Assessment of patient and provider attitudes towards therapeutic drug monitoring to improve medication adherence in low- income patients with hypertension: a qualitative study |  | None | Patients with uncontrolled hypertension with either systolic blood pressure of at least 130 mm Hg or diastolic blood pressure of at least 80 mm Hg despite antihypertensive drugs and providers in the general cardiology and internal medicine clinics. | (1) presence of hypertensive emergency (BP >180/110 mm Hg plus one of the following features: acute coronary syndrome, acute stroke, hyperten sive encephalopathy, aortic dissection or acute kidney injury), (2) history of active substance abuse such as alcohol, cocaine or narcotics, (3) uncontrolled psychi atric disorder such as schizophrenia or major depres sion based on diagnosis entered in electronic medical record, (4) pregnancy, (5) homelessness, (6) stage V Chronic kidney disease (CKD) or end stage renal disease (glomerular filtration rate |
| Wunder 2019 | Adherence to antihypertensive drug treatment in patients with apparently treatment-resistant hypertension in the INSPiRED pilot study. | 18 | None | Patients with aTRH referred for renal denervation and included in the INSPiRED pilot trial. All patients were prescribed at least three or more AHDs of different classes, preferably including a diuretic. |  |
| Pelouch 2019 | The Assessment of Serum Drug Levels to Diagnose Non-Adherence in Stable Chronic Heart Failure Patients | 81 | None | All participants were stable with set up CHF and antihypertensive medication |  |
| Hayes 2019 | Measuring adherence to therapy in apparent treatment-resistant hypertension: A feasibility study in Irish primary care | 235 | None | Patients (volunteers) who were either on four blood pressure-lowering medications or on at least three with raised blood pressure were eligible. | GPs were asked to review the names of these patients to confirm their continuing eligibility and that they were not suffering from significant morbidity, which would preclude their ability to participate, such as those with  acute oncological issues, severe psychiatric illness, or individuals who were housebound |
| deJager 2018 | Medication adherence in patients with apparent resistant hypertension: findings from the SYMPATHY trial | 98 baseline, 83 follow-up. | None | Mean daytime systolic BP ≥135 mmHg, as determined with the use of ambulatory BP measurement (ABPM), while having been prescribed three or more AHDs. | treatable secondary cause of htn. |
| vanSchoonhoven 2018 | Cost-Utility of an Objective Biochemical Measure to Improve Adherence to Antihypertensive Treatment | N/A | None | N/A | N/A |
| Sandbaumhuter 2018 | Medication adherence during laboratory workup for primary aldosteronism: Pilot study | 24 | None | We used first and second visit blood samples from patients who participated in a prospective observational study of new predictive biomarkers while being  subjected to this protocol (NCT03034265). Age >18 years, treatment with >2 antihypertensive drugs for >3 months, and a mean office BP >140/90 mmHg on the first visit (mean of three readings with a semi-automated device). | Prespecified exclusion criteria were pregnancy, chronic renal insufficiency grade ≥4, cardiac insufficiency New York Heart Association grade IV, chronic obstructive lung disease grade ≥3, liver insufficiency CHILD grade B or C, drugs significantly interfering with the RAAS or sympathetic nervous activity that could not be paused between the two visits, treatment with aliskiren or a mineralocorticoid receptor antagonist, and a BP ≥180/100 mmHg on the first visit |
| Sutherland 2018 | Assessment of Patient Medication Adherence, Medical Record Accuracy, and Medication Blood Concentrations for Prescription and Over-the-Counter Medications |  | None | Patients with hypertension treated in an adult emergency department. prescribed at least 1 of 14 antihypertensive medications for hypertension. |  |
| Avataneo 2018 | Therapeutic drug monitoring-guided definition of adherence profiles in resistant hypertension and identification of predictors of poor adherence | 50 | None | Patients with resistant hypertension (office SBP >140 mm Hg and/or office DBP > 90 mmHg, despite regular intake of maximally tolerated doses of at least three antihypertensive drugs including a diuretic for at least 6 weeks) | Secondary, white coat, drug related or manifest nonadherence were excluded. |
| Petit 2018 | Impact of psychological profile on drug adherence and drug resistance in patients with apparently treatment-resistant hypertension | 35 | None | Patients with aTRH. Most patients included were third referrals who had seen several specialists (cardiologists, nephrologists) and remained for months or years with uncontrolled BP despite complex therapeutic regimens before consulting at our Academic Hospital. |  |
| Gupta 2017 (1) | Risk Factors for Nonadherence to Antihypertensive Treatment | 1,348 | None | Main criterion for screening (and therefore inclusion in this analysis) was suspected therapeutic non-adherence. Patients with hypertension with suspected nonadherence and those with suboptimal BP control |  |
| Jones 2017 | Therapeutic drug monitoring of amlodipine and the Z-FHL/HHL ratio : adherence tools in patients referred for apparent treatment-resistant hypertension | 100 | None | Patients attending a referral hypertension clinic for apparent treatment-resistant hypertension, who were receiving both enalapril and amlodipine with additional antihypertensives, were enrolled into the study. | none stated |
| Hamdidouche 2017 | Routine urinary detection of antihypertensive drugs for systematic evaluation of adherence to treatment in hypertensive patients | 174 | None | Adult outpatients with essential hypertension referred to an academic medical center specialty hypertension clinic, prescribed at least 1 AHD. | Severe uncontrolled hypertension (BP >200 mmHg and/or DBP >130mmHg). severe reduced kidney function that may influence renal excretion of antihypertensive drugs, and serious physical or psychiatric impairment that limited ability to self-administer antihypertensive medications. did not include patients receiving only AHDs that could not be detected with UPLCMSMS method. |
| Gupta 2017 | Biochemical Screening for Nonadherence Is Associated With Blood Pressure Reduction and Improvement in Adherence | UK: 238 Czechia: 93 | None | UK: retrospective data. patients attending BP clinic who had at least one CAT during study period. CAT for nonadherence were patients suspected to deviate from the prescribed antihypertensive therapy by their managing doctor.  Czechia: patients referred with suboptimal BP control to hospital hypertension unit whose CAT showed nonadherence. The Czech patients were referred for LC-MS/MS-based analysis if their treating clinician found their BP control was suboptimal on the existing antihypertensive treatment |  |
| Kocianova 2017 | Heart rate is a useful marker of adherence to beta-blocker treatment in hypertension. | 106 (220 measurements) | None | Consecutive patients with apparently resistant arterial hypertension who had had beta-blocker levels measured to assess adherence to the recommended medical therapy. Included only patients who confirmed regular intake of antihypertensive medication including the beta blocker and also stated its use at least 2 hours before blood sampling in the morning. | Patients with atrial fibrillation, other arrhythmias or stimulated rhythm were excluded |
| McNaughton 2017 | Systolic Blood Pressure and Biochemical Assessment of Adherence: A Cross-Sectional Analysis in the Emergency Department | 261 | None | Convenience sample of adults with treated hypertension who sought ED care. |  |
| Bohlender 2017 | Medication adherence during work-up for Conn syndrome | 24 | Blood samples from first and second visit to clinic. | Patients undergoing evaluation for primary aldosteronism. |  |
| Schmieder 2016 | Adherence to antihypertensive medication in treatment-resistant hypertension undergoing renal denervation | 79 | none | Participants enrolled in clinical trials of renal denervation.    TRH (≥140/90 despite ≥3antihypertensives incl a diuretic), confirmed with ABPM. |  |
| Patel 2016 | Screening for non-adherence to antihypertensive treatment as a part of the diagnostic pathway to renal denervation | 34 | None | Patients referred for renal denervation. Patients without evidence of white-coat effect or white-coat hypertension in whom out-of-office daytime SBP was 4150 mm Hg were eligible for further diagnostic steps in the pathway for renal denervation. |  |
| Beaussier 2015 | True antihypertensive efficacy of sequential nephron blockade in patients with resistant hypertension and confirmed medication adherence | 164 | None | Patients with RH. Essential hypertension resistant to three or more AHDs, including a diuretic (supine office BP of at least 140 and/or 90 mmHg) | secondary htn, hx of severe cardiovascular disease or stroke in preceeding 3 months, atrial fibrillation, uncontrolled DM, eGFR <30ml/min. |
| Ewen 2015 | Blood pressure reductions following catheter-based renal denervation are not related to improvements in adherence to antihypertensive drugs measured by urine/plasma toxicological analysis | 100 | None (baseline and 6 months post RDN) | Consecutive patients undergoing bilateral RDN. Patients with resistant hypertension, defined as baseline office systolic blood pressure >=140 mmHg despite treatment with >=3 antihypertensive agents with stable antihypertensive regimen who completed 6 month follow-up.   Patients were asked whether they had taken their current medication. "After a positive declaration, plasma and urine samples were obtained at baseline and at 6-month follow-up". | secondary, treatable causes of hypertension were excluded |
| Florczak 2015 | Assessment of adherence to treatment in patients with resistant hypertension using toxicological serum analysis | 36 | None | Good adherence as judged by primary care physician. Patients hospitalised for purpose of CAT. Main study (RESIST-POL) included patients with resistant hypertension confirmed by ABPM, preserved renal function, and no history of diabetes   Subgroup for CAT: patients who met the following 3 criteria: antihypertensive regimen of at least 4 drugs, average daytime ambulatory systolic BP of 140 mmHg or higher, and one of the clinical features that may suggest nonadherence during the study (eg, tachycardia while using an adequate dose of β-blocker or lower potassium plasma levels when taking spironolactone) | Secondary causes of HTN. |
| Velasco 2015 | Cost-Effectiveness of Therapeutic Drug Monitoring in Diagnosing Primary Aldosteronism in Patients With Resistant Hypertension | 78 of 227 patients underwent CAT. | None | Patients referred to hypertension specialist clinic for aTRH. All patients reported that they were adherent to all antihypertensive medications prior to TDM | Patients were excluded if they were intolerant to three or more antihypertensive drug classes. Screen- ing for white-coat effect with 24-hour ambulatory BP monitoring was conducted for patients who reported normal home BP (<135/85 mm Hg), and patients with demonstrated BP control at home were also excluded |
| Tomaszewski 2014 | High rates of non-adherence to antihypertensive treatment revealed by high-performance liquid chromatography-tandem mass spectrometry (HP LC-MS/MS) urine analysis | 208 | None | Patients (i)consecutive, unselected newly referred to a specialist htn centre (ii) already attending a specialist hypertension centre with inadequate BP control (iii) referred for renal denervation |  |
| Rosa 2014 | Importance of thorough investigation of resistant hypertension before renal denervation: should compliance to treatment be evaluated systematically?. | 205 |  | Patients were referred to a Hypertension Centre. The reason of referral (based on the decision referring physician) was not specifically the question of possible renal denervation but severe or difficult to treat hypertension and/or suspicion of secondary cause of hypertension. |  |
| Brinker 2014 | Therapeutic drug monitoring facilitates blood pressure control in resistant hypertension | 56 | None | Patients attending hospital clinic who met the definition of RH |  |
| Jung 2013 | Resistant hypertension? Assessment of adherence by toxicological urine analysis | 76 | None | Patients with unexplained apparent resistant hypertension (taking at least four antihypertensive drugs; in whom secondary hypertension was excluded). LC-MS analysis was performed only when patients had previously confirmed regular drug intake during the last days prior to clinical visit. |  |
| Strauch 2013 | Precise assessment of noncompliance with the antihypertensive therapy in patients with resistant hypertension using toxicological serum analysis | 339 | None | (i) Patients admitted to hospital for exclusion of secondary cause for hypertension. (ii)Outpatients investigated for the first time in an outpatient hypertension clinic. Included only patients who confirmed regular drug intake before the visit/hospitalization | Patients, in whom we later confirmed secondary hypertension (e.g. primary aldosteronism, pheochromocytoma, hypercortisolism, significant renal artery stenosis, renal parenchymal hypertension and obstructive sleep apnoea), were not included in this study |
| Ceral 2011 | Difficult-to-control arterial hypertension or uncooperative patients the assessment of serum antihypertensive drug levels to differentiate non-responsiveness from non-adherence to recommended therapy | 84 | None | Consecutive patients with difficult-to control arterial hypertension. Included if they stated that they had taken drug the evening before or day of their clinic visit. |  |
| Azizi 2006 | Assessment of Patients' and Physicians' Compliance to an ACE Inhibitor Treatment Based on Urinary N-Acetyl Ser-Asp-Lys-Pro Determination in the Noninsulin-Dependent Diabetes, Hypertension, Microalbuminuria, Proteinuria, Cardiovascular Events, and Ramipril | 1871 | Trial compared low-dose ramipril with placebo in patients with diabetes and microalbuminuria | Proteinuric or microalbuminuric type 2 diabetes patients |  |
