# Supplementary material for: Chemical adherence testing in the clinical management of hypertension: a scoping review
Source: Front Pharmacol. 2024 Nov 6;15:1452464. doi: 10.3389/fphar.2024.1452464 (PMC11576289; doi:10.3389/fphar.2024.1452464)
Supplement: Supplementary file 3 [file DataSheet1.pdf]

Ovid®

My Account | My PayPerView | Support & Training | Help | Feedback | Log Off | Louise Rabbitt

Search | Journals | Books | My Workspace | Visible Body | Multimedia

Search History (16)

View Saved

#	Searches	Results	Type	Actions		Annotations
16	3 and 10 and 15	179	Advanced	Display Results	More	
15	11 or 12 or 13 or 14	821519	Advanced	Display Results	More	
14	(hypertens* or antihypertens* or anti-hypertens*).mp. [mp=title, book title, abstract, original title, name of substance word, subject heading word, floating sub-heading word, keyword heading word, organism supplementary concept word, protocol supplementary concept word, rare disease supplementary concept word, unique identifier, synonyms, population supplementary concept word, anatomy supplementary concept word]	637625	Advanced	Display Results	More	
13	Antihypertensive Drugs/	0	Advanced	Save	More	
12	Blood Pressure/	294526	Advanced	Display Results	More	
11	exp Hypertension/	323699	Advanced	Display Results	More	
10	4 or 5 or 6 or 7 or 8 or 9	138318	Advanced	Display Results	More	
9	Mass Spectrometry/	108518	Advanced	Display Results	More	
8	therapeutic drug monitoring.mp. [mp=title, book title, abstract, original title, name of substance word, subject heading word, floating sub-heading word, keyword heading word, organism supplementary concept word, protocol supplementary concept word, rare disease supplementary concept word, unique identifier, synonyms, population supplementary concept word, anatomy supplementary concept word]	11483	Advanced	Display Results	More	
7	Drug Monitoring/	23816	Advanced	Display Results	More	
6	(medication adj3 compliance adj3 test$3).mp. [mp=title, book title, abstract, original title, name of substance word, subject heading word, floating sub-heading word, keyword heading word, organism supplementary concept word, protocol supplementary concept word, rare disease supplementary concept word, unique identifier, synonyms, population supplementary concept word, anatomy supplementary concept word]	22	Advanced	Display Results	More	
5	(medication adj3 adherence adj3 test*).mp. [mp=title, book title, abstract, original title, name of substance word, subject heading word, floating sub-heading word, keyword heading word, organism supplementary concept word, protocol supplementary concept word, rare disease supplementary concept word, unique identifier, synonyms, population supplementary concept word, anatomy supplementary concept word]	117	Advanced	Display Results	More	
4	(chemical adj3 adherence adj3 test*).mp. [mp=title, book title, abstract, original title, name of substance word, subject heading word, floating sub-heading word, keyword heading word, organism supplementary concept word, protocol supplementary concept word, rare disease supplementary concept word, unique identifier, synonyms, population supplementary concept word, anatomy supplementary concept word]	7	Advanced	Display Results	More	
3	1 or 2	130249	Advanced	Display Results	More	
2	((drug* or medication* or therap* or treatment* or anithypertens* or anti-hypertens* or chemical* or biochemical*) adj3 (adherence or compliance or persistence or nonadherence or non-adherence)).mp.	81506	Advanced	Display Results	More	
1	"treatment adherence and compliance"/ or patient compliance/ or medication adherence/	85985	Advanced	Display Results	More	

Save | Remove | Combine with: AND | OR | Contract

Save All | Edit | Create RSS | Create Auto-Alert | View Saved | Share Search History

https://ovidsp.ovid.com/ovidweb.cgi?T=JS&NEWS=N&PAGE=main&SHAREDSEARCHID=6biWSMw7uUQHHP9PqtKZirz8EDMueUpKfxDLcwM9HwfdXXfvkS6L3mK9gIg1tuBFt

Ovid MEDLINE(R) ALL <1946 to April 10, 2024>

1 "treatment adherence and compliance"/ or patient compliance/ or medication adherence/ 85985

2 ((drug* or medication* or therap* or treatment* or anithypertens* or anti-hypertens* or chemical* or biochemical*) adj3 (adherence or compliance or persistence or nonadherence or non-adherence)).mp. 81506

3 1 or 2 130249

4 (chemical adj3 adherence adj3 test*).mp. [mp=title, book title, abstract, original title, name of substance word, subject heading word, floating sub-heading word, keyword heading word, organism supplementary concept word, protocol supplementary concept word, rare disease supplementary concept word, unique identifier, synonyms, population supplementary concept word, anatomy supplementary concept word] 7

5 (medication adj3 adherence adj3 test*).mp. [mp=title, book title, abstract, original title, name of substance word, subject heading word, floating sub-heading word, keyword heading word, organism supplementary concept word, protocol supplementary concept word, rare disease

supplementary concept word, unique identifier, synonyms, population supplementary concept word, anatomy supplementary concept word] 117

6 (medication adj3 compliance adj3 test$3).mp. [mp=title, book title, abstract, original title, name of substance word, subject heading word, floating sub-heading word, keyword heading word, organism supplementary concept word, protocol supplementary concept word, rare disease supplementary concept word, unique identifier, synonyms, population supplementary concept word, anatomy supplementary concept word] 22

7 Drug Monitoring/ 23816

8 therapeutic drug monitoring.mp. [mp=title, book title, abstract, original title, name of substance word, subject heading word, floating sub-heading word, keyword heading word, organism supplementary concept word, protocol supplementary concept word, rare disease supplementary concept word, unique identifier, synonyms, population supplementary concept word, anatomy supplementary concept word] 11483

9 Mass Spectrometry/ 108518

10 4 or 5 or 6 or 7 or 8 or 9 138318

11 exp Hypertension/ 323699

12 Blood Pressure/ 294526

13 Antihypertensive Drugs/ 0

14 (hypertens* or antihypertens* or anti-hypertens*).mp. [mp=title, book title, abstract, original title, name of substance word, subject heading word, floating sub-heading word, keyword heading word, organism supplementary concept word, protocol supplementary concept word, rare disease supplementary concept word, unique identifier, synonyms, population supplementary concept word, anatomy supplementary concept word] 637625

15 11 or 12 or 13 or 14 821519

16 3 and 10 and 15 179
