# Supplementary material for: Chemical adherence testing in the clinical management of hypertension: a scoping review
Source: Front Pharmacol. 2024 Nov 6;15:1452464. doi: 10.3389/fphar.2024.1452464 (PMC11576289; doi:10.3389/fphar.2024.1452464)
Supplement: Supplementary file 4 [file Table3.docx]

Supplemental Table 3 – Judgements according to EMERGE minimum reporting criteria

| Author, year | Year | Country | Setting | Primary aim of study | Study design | Total number of participants |
| --- | --- | --- | --- | --- | --- | --- |
| Peeters 2024 | 2024 | Netherlands | Vascular, cardiology and nephrology hospital departments | To determine whether a CAT intervention combined with feedback using a communication tool leads to a decrease in resistant hypertension. | RCT | 100 |
| Kario 2023 | 2023 | Japan | Clinical trial | Post-hoc analysis of stored urine samples in order to evaluate medication adherence. | Post-hoc analysis within RCT | 58 |
| Kustovs 2023 | 2023 | Latvia | University hospital | To establish a target population of patients with possible changes in drug compliance despite the wide range of fixed-dose combinations and in whom it would be useful to determine the concentration of amlodipine in the blood. | Prospective cross sectional study | 81 |
| Seleznev 2023 | 2023 | Russia | Regional Clinical Cardiological Dispensary | To test the concentration of antihypertensive drugs in patients with uncontrolled and controlled arterial hypertension | Cohort study | 46 |
| Curneen 2023 | 2023 | Ireland | Specialist hypertension clinic | To compare patient reported antihypertensive adherence with objective evidence using mass spectrometry spot urinalysis. | Prospective cohort study | 73 |
| Peeters 2023 | 2023 | Netherlands | Hospital nephrology and vascular clinics | To determine the adherence to antihypertensive drugs in patients visiting the nephrology and vascularoutpatient clinics using CAT. | Prospective cross sectional study | 142 |
| Osman 2023 | 2023 | UK | University hospital renal clinic | To demonstrate and highlight the usefulness of CAT to determine the prevalence of nonadherence to cardio-metabolic medications in patients attending routine renal clinics. | Prospective cross sectional study | 106 |
| Bourque 2023 | 2023 | Canada | Multiple | To report on the overall prevalence of nonadherence in the apparent treatment resistant hypertension population and the quantitative contributions to nonadherence based on different methods of assessment, with an emphasis on attempting to explain the heterogeneity of the data. | Systematic review and meta-analysis | 71,353 |
| Georges 2022 | 2022 | Belgium; Italy | Cardiology Dept; Hypertension Expert Centre | To document associations between psychological profile, drug adherence, and severity of hypertension in a representative sample of patients with apparent treatment resistant hypertension, using controlled hypertensive patients as the comparator. | Prospective cross sectional Study. | 144 |
| Sheppard 2022 | 2022 | UK | Primary care | To investigate whether it is feasible to collect urine samples in a primary care setting and analyse them using the LC-MS/MS method to measure adherence to antihypertensive medications. | Prospective cohort study | 191 |
| Groenland 2022 | 2022 | Netherlands; UK | Hospital outpatient clinics | To develop and externally validate a screening tool, based on easy to collect clinical variables, to estimate the probability of non-adherence in patients with uncontrolled hypertension. | Cross sectional study | 735 |
| Peeters 2022 | 2022 | Netherlands | Clinical trial | To illustrate the importance and difficulties that can arise using a three-step approach to medication adherence . | Case series within RCT | 3 |
| Osula 2022 | 2022 | United States | Internal Medicine and Cardiology Clinics in a large  urban safety net health system | To compare the sensitivity, specificity, and predictive values of pharmacy fill data measures of adherence obtained from a nationwide prescribing database against CAT in detecting nonadherence with cardiovascular medications in patients with uncontrolled hypertension in the safety net health system. | Prospective cross sectional study | 77 |
| Wang 2021 | 2021 | China | Hospital | To ensure drug compliance during a catheter-based therapy for treatment of hypertension. | Cross sectional study | 92 |
| Buffolo 2021 | 2021 | Italy | Hypertension unit of university hospital | To evaluate the aldosterone:renin ratio changes, before and after ARB/ACEi initiation, as a means to assess adherence to ARB/ACEi prescription. | Prospective cohort study | 40 |
| Beernink 2021 | 2021 | Netherlands | Hospital/Trial | To assess the prevalence of nonadherence to oral antidiabetics, antihypertensives, and statins within a cohort study of type 2 diabetes patients managed in a specialist setting using CAT. | Prospective cohort study | 457 |
| Schäfer 2021 | 2021 | Germany | Hypertension clinic in university medical centre | To analyse patients' suitability for baroreceptor activation therapy and reasons for non-eligibility in patients with apparently resistant hypertension. | Retrospective cross sectional study | 75 |
| Lauder 2021 | 2021 | Germany | Emergency Department of University Medical Centre | To identify treatment-related and psychosocial characteristics, including anxiety, depression, and health literacy, associated with nonadherence to BP-lowering medication among patients with previously diagnosed hypertension presenting with hypertensive urgencies at an emergency department. | Prospective cross sectional study | 104 |
| Schesing 2020 | 2020 | USA | Outpatient clinics in an integrated health system which provides care for a low- income, uninsured population | To explore patients' and providers' knowledge, attitudes, beliefs and concerns about using a blood test to monitor medication adherence and how best to introduce and use CAT in a respectful, patient-centred way. | Qualitative study | 21 |
| Wunder 2019 | 2019 | Belgium, Netherlands | Clinical trial | To give an impression on the reliability of adherence assessment during a trial. | Analysis within randomised parallel group trial | 18 |
| Pelouch 2019 | 2019 | Czechia | Hospital clinic | To assess the drug non-adherence in stable CHF patients using serum drug levels monitoring. | Prospective cross sectional study | 81 |
| Hayes 2019 | 2019 | Ireland | Primary care | To examine the feasibility of establishing non-adherence to medication using mass spectrometry urine analysis in primary care. | Prospective cross sectional study | 235 |
