# Supplementary material for: Chemical adherence testing in the clinical management of hypertension: a scoping review
Source: Front Pharmacol. 2024 Nov 6;15:1452464. doi: 10.3389/fphar.2024.1452464 (PMC11576289; doi:10.3389/fphar.2024.1452464)
Supplement: Supplementary file 5 [file Table4.docx]

Supplemental Table 4: Judgements for JBI tool for assessing quality of cross-sectional studies

| Author, year | Study design | Were the criteria for inclusion in the sample clearly defined? | Were the study subjects and the setting described in detail? | Was the exposure measured in a valid and reliable way? | Were objective, standard criteria used for measurement of the condition? | Were confounding factors identified? | Were strategies to deal with confounding factors stated? | Were the outcomes measured in a valid and reliable way? | Was appropriate statistical analysis used? |
| --- | --- | --- | --- | --- | --- | --- | --- | --- | --- |
| Kustovs 2023 | Prospective cross sectional study | yes | no | yes | no | no | no | Unsure | yes |
| Seleznev 2023 | Cohort study | no | no | no | no | no | no | no | no |
| Curneen 2023 | Prospective cohort study | yes | yes | N/A | yes | yes | yes | yes | yes |
| Peeters 2023 | Prospective cross sectional study | yes | yes | N/A | yes | yes | no | yes | yes |
| Osman 2023 | Prospective cross sectional study | yes | yes | N/A | Unsure | yes | yes | yes | yes |
| Georges 2022 | Prospective cross sectional Study. | yes | yes | N/A | yes | yes | yes | yes | yes |
| Sheppard 2022 | Prospective cohort study | yes | yes | N/A | yes | yes | Unsure | Unsure | yes |
| Groenland 2022 | Cross sectional study | yes | yes | N/A | yes | yes | yes | yes | yes |
| Osula 2022 | Prospective cross sectional study | yes | yes | N/A | yes | yes | no | yes | yes |
| Wang 2021 | Cross sectional study | no | no | N/A | yes | yes | no | Unsure | Unsure |
| Buffolo 2021 | Prospective cohort study | yes | yes | yes | yes | no | no | yes | yes |
| Beernink 2021 | Prospective cohort study | yes | yes | N/A | yes | yes | yes | yes | yes |
| Schäfer 2021 | Retrospective cross sectional study | no | yes | N/A | yes | yes | no | yes | Unsure |
| Lauder 2021 | Prospective cross sectional study | yes | yes | N/A | yes | yes | no | yes | yes |
| Wunder 2019 | Analysis within randomised parallel group trial | yes | yes | N/A | yes | yes | Unsure | yes | Unsure |
| Pelouch 2019 | Prospective cross sectional study | Unsure | Unsure | N/A | Unsure | yes | no | yes | Unsure |
| Hayes 2019 | Prospective cross sectional study | yes | yes | N/A | yes | yes | no | yes | yes |
| deJager 2018 | Substudy of open label RCT | yes | yes | N/A | yes | yes | yes | yes | yes |
| Sandbaumhüter 2018 | Prospective cohort study | yes | Unsure | N/A | yes | yes | Unsure | yes | yes |
| Sutherland 2018 | Prospective cross sectional study | yes | yes | N/A | yes | Unsure | Unsure | yes | yes |
| Avataneo 2018 | Prospective cross-sectional. | yes | yes | N/A | yes | yes | yes | yes | yes |
| Petit 2018 | Prospective cross sectional study | yes | yes | N/A | yes | yes | Unsure | yes | yes |
| Gupta 2017 (1) | Retrospective cross sectional study. | no | no | N/A | yes | yes | Unsure | yes | yes |
| Jones 2017 | Prospective cross sectional study | no | Unsure | N/A | yes | no | no | yes | yes |
| Hamdidouche 2017 | Prospective cohort study | yes | yes | yes | yes | yes | yes | yes | yes |
| Gupta 2017 (2) | Retrospective cohort study | yes | yes | yes | yes | yes | no | yes | yes |
| Kocianova 2017 | Retrospective cross sectional study | Unsure | yes | yes | yes | Unsure | Unsure | yes | yes |
| McNaughton 2017 | Prospective cross-sectional study | yes | yes | N/A | yes | yes | yes | yes | yes |
| Bohlender 2017 | Prospective observational pilot study | yes | yes | N/A | yes | yes | yes | yes | Unsure |
| Schmieder 2016 | Analysis within prospective clinical trial | yes | yes | yes | yes | yes | no | yes | yes |
| Patel 2016 | Retrospective analysis | yes | yes | N/A | yes | yes | Unsure | yes | yes |
| Ewen 2015 | Prospective cohort study | yes | yes | yes | yes | yes | yes | yes | yes |
| Florczak 2015 | Cross sectional study | no | yes | N/A | yes | no | no | yes | yes |
| Velasco 2015 | Cross sectional study; Economic Evaluation | yes | yes | N/A | yes | Unsure | Unsure | yes | yes |
| Tomaszewski 2014 | Retrospective cross sectional study | yes | yes | yes | yes | yes | no |  | yes |
| Rosa 2014 | Cohort study | yes | yes | Unsure | yes | yes | Unsure | yes | yes |
| Brinker 2014 | Retrospective study | yes | no | N/A | no | no | no | yes | Unsure |
| Jung 2013 | Retrospective chart review | yes | yes | yes | yes | yes | yes | yes | yes |
| Strauch 2013 | Cohort study | yes | yes | N/A | yes | yes | yes | yes | yes |
| Ceral 2011 | Retrospective cross sectional study | yes | no | N/A | yes | no | no | yes | yes |
